# Pharmacokinetic/pharmacodynamic modeling of ketoprofen and flunixin at piglet castration and tail‐docking

**DOI:** 10.1111/jvp.13083

**Published:** 2022-07-14

**Authors:** Emma Nixon, Jason T. Chittenden, Ronald E. Baynes, Kristen M. Messenger

**Affiliations:** ^1^ Department of Population Health and Pathobiology, College of Veterinary Medicine North Carolina State University Raleigh North Carolina USA; ^2^ Regeneron Pharmaceuticals, Inc. Tarrytown New York USA; ^3^ Department of Molecular Biomedical Sciences, College of Veterinary Medicine North Carolina State University Raleigh North Carolina USA

**Keywords:** NSAID, pain, pharmacodynamics, pharmacokinetics, piglet

## Abstract

This study performed population‐pharmacokinetic/pharmacodynamic (pop‐PK/PD) modeling of ketoprofen and flunixin in piglets undergoing routine castration and tail‐docking, utilizing previously published data. Six‐day‐old male piglets (8/group) received either ketoprofen (3.0 mg/kg) or flunixin (2.2 mg/kg) intramuscularly. Two hours post‐dose, piglets were castrated and tail docked. Inhibitory indirect response models were developed utilizing plasma cortisol or interstitial fluid prostaglandin E2 (PGE2) concentration data. Plasma IC50 for ketoprofen utilizing PGE2 as a biomarker was 1.2 μg/ml, and ED50 for was 5.83 mg/kg. The ED50 calculated using cortisol was 4.36 mg/kg; however, the IC50 was high, at 2.56 μg/ml. A large degree of inter‐individual variability (124.08%) was also associated with the cortisol IC50 following ketoprofen administration. IC50 for flunixin utilizing cortisol as a biomarker was 0.06 μg/ml, and ED50 was 0.51 mg/kg. The results show that the currently marketed doses of ketoprofen (3.0 mg/kg) and flunixin (2.2 mg/kg) correspond to drug responses of 33.97% (ketoprofen‐PGE2), 40.75% (ketoprofen‐cortisol), and 81.05% (flunixin‐cortisol) of the maximal possible responses. Given this information, flunixin may be the best NSAID to use in mitigating castration and tail‐docking pain at the current label dose.

## 1 INTRODUCTION

In the United States, piglets are subjected to routine yet painful husbandry procedures such as castration and tail‐docking without analgesia. In the EU and Canada, certain nonsteroidal anti‐inflammatory drugs (NSAIDs) are approved to reduce pain associated with castration; however, there are no FDA‐approved medications indicated to treat pain in pigs in the United States.

Several studies have assessed the effectiveness of NSAIDs to reduce pain associated with castration; however, there are conflicting results regarding their analgesic efficacy, and pharmacodynamic effects are highly variable (Bates et al., 2014; Dzikamunhenga et al., 2014; Keita et al., 2010; Kluivers‐Poodt et al., 2013; Sutherland et al., 2012; Tenbergen et al., 2014; Viscardi & Turner, 2018). In addition, there is limited knowledge regarding the optimal dose required to provide effective analgesic and anti‐inflammatory effects.

The primary mechanism of action for NSAIDs is inhibition of the cyclooxygenase (COX) enzyme. The COX‐2 isoform is inducible and upregulated by tissue damage and inflammatory stimuli, increasing production of prostaglandins, and prostaglandin E2 (PGE2) increases following castration and tail docking in piglets (Fosse et al., 2008; Nixon et al., 2021). Prostaglandins contribute to pain signaling by activating and sensitizing nociceptors (Davidson et al., 2014), leading to an increase in the magnitude of response to noxious stimulation. Inhibition of the COX‐2 isoform is the most likely mechanism for NSAID‐mediated analgesia (Cashman, 1996); therefore, reduction in PGE2 should decrease nociception following castration and tail‐docking (Bates et al., 2014).

In response to stress, corticotrophin‐releasing hormone (CRH) is released by the hypothalamus, stimulating the secretion of adrenocorticotrophic hormone (ACTH) from the anterior pituitary which acts on the adrenal gland to produce cortisol. Prostaglandins also directly stimulate ACTH and cortisol release (Sheil et al., 2020), and piglet castration research commonly uses cortisol as an indirect measure of pain (Bates et al., 2014; Ison et al., 2016; Keita et al., 2010; Kluivers‐Poodt et al., 2012; Nixon et al., 2021; Prunier et al., 2005; Sutherland et al., 2011). A panel of experts performed a systematic review of available data related to pain mitigation during piglet husbandry procedures. Most of the panel decisions relied primarily on cortisol as an outcome, leading to a weak recommendation for the use of NSAIDs (O'Connor et al., 2014).

Several PK/PD models are published that describe the effect of NSAIDs on pain and inflammation in other veterinary species, and IC50 values (specifically describing PGE2 suppression) have been generated for NSAIDs in other species as well (Table 1). However, there are relatively few PK/PD models for NSAIDs in pigs (Fosse, Toutain, et al., 2011; Levionnois et al., 2018). Both previously published PK/PD models for NSAIDs in pigs were developed via an induced inflammation model rather than routine castration, and these models also did not establish IC50 values for suppression of PGE2. In addition, studies have suggested that plasma drug concentrations do not always reflect tissue drug concentrations, particularly for NSAIDs, which may become “trapped” at sites of inflammation (Brune & Furst, 2007; Lees et al., 2004; Messenger et al., 2016). The previous portion of this study shows that NSAIDs given before processing procedures may reduce pain and inflammation associated with castration and tail docking (Nixon et al., 2021), and interstitial fluid (ISF) drug concentrations were also previously reported (Nixon et al., 2020). Interstitial fluid collected via in vivo ultrafiltration allows the measurement of only the pharmacologically active, protein‐unbound drug concentrations, critical to assess drug concentrations directly at the tissue level and may better correlate with the anti‐inflammatory effect than plasma concentrations. The authors are not currently aware of any PK/PD models that assess the impact of NSAIDs on cortisol concentrations in any species, and there are no reported IC50 values specifically describing the effect on cortisol.

**TABLE 1:** Overview of flunixin and ketoprofen (racemic and S‐[+]‐ketoprofen) pharmacodynamic parameters collected from existing literature for various species, where PGE2 inhibition was the outcome of interest

Species	IC_50_	IC_80_	Imax	Source
	μg/ml	μg/ml	%	
Flunixin				
Alpaca	–	0.230	–	(Reppert et al., 2019)
Calf	–	0.026 (pain)	–	(Kleinhenz et al., 2018)
	–	0.039 (no pain)	–	(Kleinhenz et al., 2018)
	0.009^a^	0.049^a^	123.00	(Miciletta et al., 2014)
Horse	0.033^a^	–	–	(Marshall, 2010)
	0.063^b^	0.895^b^	–	(Beretta et al., 2005)
	0.019	–	109.04	(Landoni & Lees, 1995a)
	0.053^a^	–	–	(Brideau et al., 2001)
Racemic ketoprofen				
Calf	0.086	–	–	(Landoni et al., 1995)
Cat	0.046	–	–	(Pelligand et al., 2014)
Dog	0.06^a^	–	–	(Brideau et al., 2001)
Goat	0.028	–	112.00	(Arifah et al., 2003)
Horse	0.057	–	100.88	(Landoni & Lees, 1995a)
Sheep	0.012	–	92.00	(Landoni et al., 1999)
S‐(+)‐ketoprofen				
Calf	0.042	–	99.00	(Landoni & Lees, 1995b)
Goat	0.003	–	100.00	(Arifah et al., 2003)
Horse	0.033	–	–	(Landoni & Lees, 1996)
Sheep	0.007	–	94.00	(Landoni et al., 1999)

^a^ Converted units from μM.

^b^ Converted units from (−log M).

The objective of this study was to study the anti‐inflammatory and analgesic effects of flunixin and ketoprofen at piglet castration and tail‐docking and to establish critical pharmacodynamic parameters for these effects via pop‐PK/PD modeling.

## 2 METHODS

### 2.1 The data source for model development

The data used in developing these models were previously published (Nixon et al., 2020, 2021). Briefly, 6‐day‐old male piglets (8/group) received one of five randomized treatments: intramuscular saline, meloxicam (0.4 mg/kg), flunixin (2.2 mg/kg), ketoprofen (3.0 mg/kg), or sham (saline injection, no processing). Two hours post‐dose, piglets were castrated and tail docked. The previous report examined various efficacy measures; however, these models only utilize plasma cortisol or interstitial fluid PGE2 concentration data. Meloxicam was excluded from the PK/PD modeling for two reasons; (1) the PK/PD models for meloxicam did not pass the validation process, (2) meloxicam was the least effective of the three NSAIDs overall, and so it is more useful to focus on flunixin and ketoprofen. The flunixin‐PGE2 model was also excluded from the PK/PD modeling due to an inability to pass the validation process.

### 2.2 Sample analysis

Plasma and ISF drug, plasma cortisol and ISF PGE2 were analyzed as previously reported (Nixon et al., 2020, 2021). In brief, plasma S‐(+)‐ketoprofen concentrations were determined by high‐performance liquid chromatography (HPLC) with UV detection, with a LOQ of 0.05 μg/ml, accuracy of 101 ± 4% and precision of 7 ± 5%. ISF S‐(+)‐ketoprofen concentrations were determined by ultra‐high‐pressure liquid chromatography (UPLC) with tandem mass spectromic (MS/MS) detection, with a LOQ of 0.001 μg/ml, accuracy of 101 ± 11%, and precision of 7 ± 6%. Plasma flunixin concentrations were determined by UPLC‐MS/MS, with a LOQ of 0.0005 μg/ml, accuracy of 103 ± 7% and precision of 8 ± 5%. ISF flunixin concentrations were determined by UPLC‐MS/MS, with a LOQ of 0.0005 μg/ml, accuracy of 100 ± 8%, and precision of 3 ± 2%.

Plasma cortisol samples were analyzed in triplicate using a commercial radioimmunoassay (RIA) kit (ImmunoChem™ Cortisol Coated Tube RIA kit, MP Biomedicals, LLC., CA, USA). Calibration curves were within the range of 1.0–25.0 μg/dl, and all *R* ^2^ values were > 0.9970. The inter‐day assay variability was 2.95 ± 1.15%, and the intra‐day assay variability was 7.88 ± 8.36%.

ISF PGE2 samples were analyzed in duplicate using a commercially available enzyme‐linked immunosorbent assay (ELISA) kit (Cayman Chemical, Co., Ann Arbor, MI, USA). *R* ^2^ for all calibration curves were > 0.96 and within the range of 7.81–1000 pg/ml. The inter‐day assay variability was 10.8%, and the intra‐day assay variability was 3.1%. All samples were analyzed in duplicate.

### 2.3 Population‐pharmacokinetic/pharmacodynamic analysis

Sequential analysis of the pharmacokinetic (PK) and pharmacodynamic (PD) data was performed using a population modeling approach with Phoenix® NLME (Version 8.3, Certara, St. Louis, MO), using first‐order conditional estimation with the extended least squares algorithm. The PK model was built first to describe the time course of NSAID concentrations in plasma. Subsequently, PD modeling analysis of the anti‐inflammatory effect of NSAIDs was performed.

The optimal model was chosen by comparison of the fits (observed vs. predicted data), the coefficient of variation (CV%) of parameter estimates, and the values of the Akaike Information Criterion and the Bayesian Information Criterion. The fitted parameters were assumed to be log‐normally distributed. The stability and performance of the final pop‐PK/PD models were assessed by assessment of visual predictive checks, and a bootstrap method performed in Phoenix NLME. Bootstrap resampling was repeated 100 times, and the values of the parameters were compared with those collected from the original dataset.

### 2.4 The pharmacokinetic models

Several alternative pop‐PK models (e.g., single compartment, different residual error models, and parameterization by clearance) were tested and discarded due to inferior performance before the selection of the final pop‐PK base model. Secondary parameter estimates were collected using standard compartmental Equations (Gabrielsson & Weiner, 2006). All models utilized a diagonal omega matrix.

### 2.5 Ketoprofen

**FIGURE 1:**
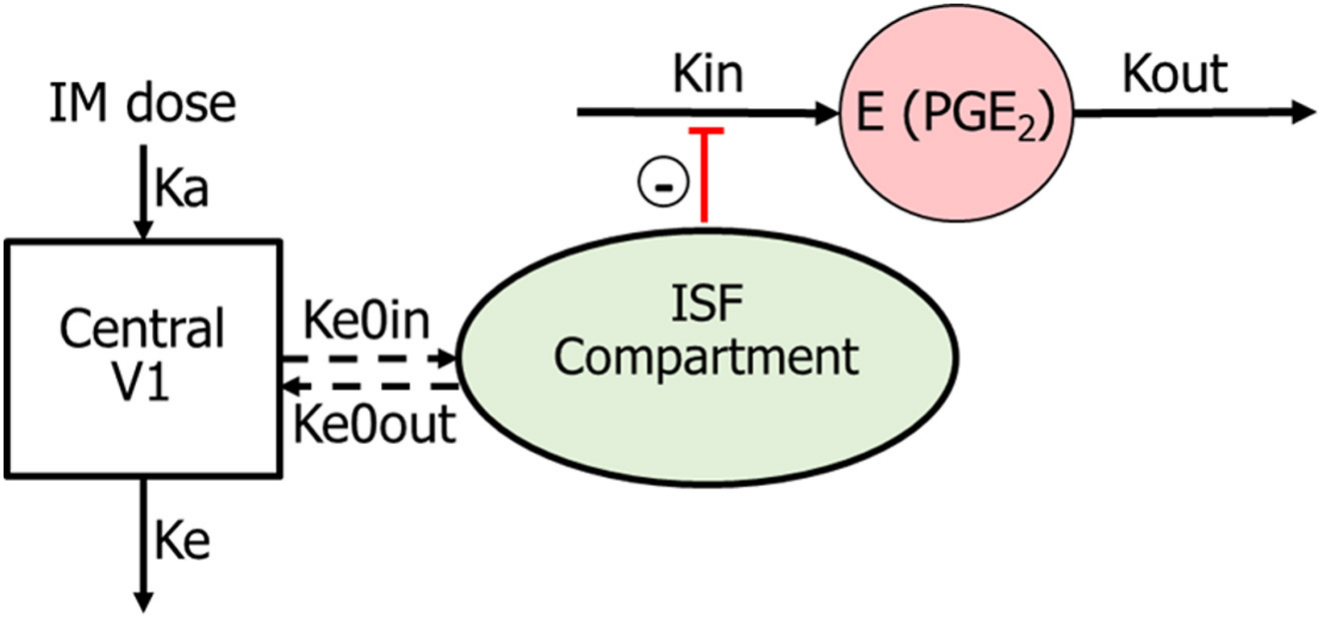
Schematic representation of the selected pharmacokinetic‐pharmacodynamic model for ketoprofen‐PGE2. Following intramuscular administration, ketoprofen is absorbed into the central compartment with a first‐order absorption rate constant ka, with drug movement between the central and ISF compartments (rate constants Ke0in and Ke0out) and is eliminated with elimination constant, Ke. In the absence of the drug, elevated PGE2 concentration results from the balance of the PGE2 production (with zero‐order rate constant kin) and PGE2 removal (with first‐order rate constant Kout). Ketoprofen that reaches the ISF induces an indirect anti‐inflammatory effect by inhibiting PGE2 production (reduction of kin)

**FIGURE 2:**
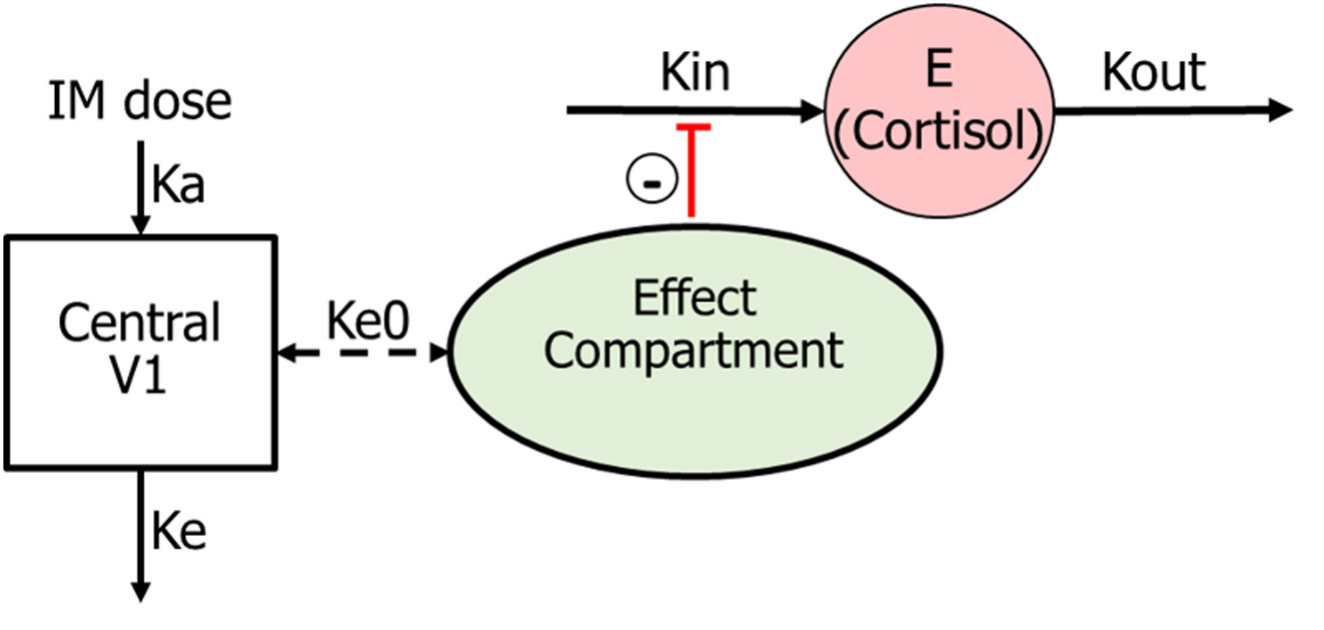
Schematic representation of the selected pharmacokinetic‐pharmacodynamic model for ketoprofen‐cortisol. Following intramuscular administration, ketoprofen is absorbed into the central compartment with a first‐order absorption rate constant ka, with drug movement between the central and effect compartments (rate constant Ke0) and is eliminated with elimination constant, Ke. In the absence of the drug, elevated cortisol concentration results from the balance of cortisol production (with zero‐order rate constant kin) and cortisol removal (with first‐order rate constant Kout). Ketoprofen induces an indirect reduction of cortisol (reduction of kin)

For ketoprofen, the S‐(+)‐enantiomer is a much more potent COX inhibitor (Suesa et al., 1993), and S‐(+)‐ketoprofen plasma concentration predominates over R‐(−)‐ketoprofen following intramuscular administration of racemic ketoprofen in piglets (Fosse, Horsberg, et al., 2011; Nixon et al., 2020). Therefore, the pop‐PK/PD modeling in this study utilized only the S‐(+)‐ketoprofen concentration data. The final model used for the pop‐PK analysis of S‐(+)‐ketoprofen (see Figures 1 and 2) assumed first‐order kinetics of absorption following intramuscular administration, one‐compartmental disposition, and micro constant parameterization. Inter‐individual variability (variance of a parameter among different subjects, or random effects) was expressed using an exponential model according to the equation:

(1) $$\mathrm{Pi}=\mathrm{\theta P}*\exp\left(\mathrm{\eta iP}\right)$$

where *Pi* is the parameter of interest for the individual i, *θP* is the population estimate for the parameter of interest, and *ηiP* is the *η* for the individual and parameter of interest. The *η* values were assumed to be independent and have a normal distribution with a mean of zero and a variance of ω^2^. A multiplicative model was used to describe the residual random variability (ε) of the data for the plasma concentrations, where ε is the residual variability with a mean of zero and a variance of σ2, according to the equation:

(2) $$\text{CObs}=\text{Cpred}*\left(1+\varepsilon \right)$$

where *CObs* is the observed plasma drug concentration for the individual and *Cpred* is the model predicted plasma drug concentration.

### 2.6 Flunixin

**FIGURE 3:**
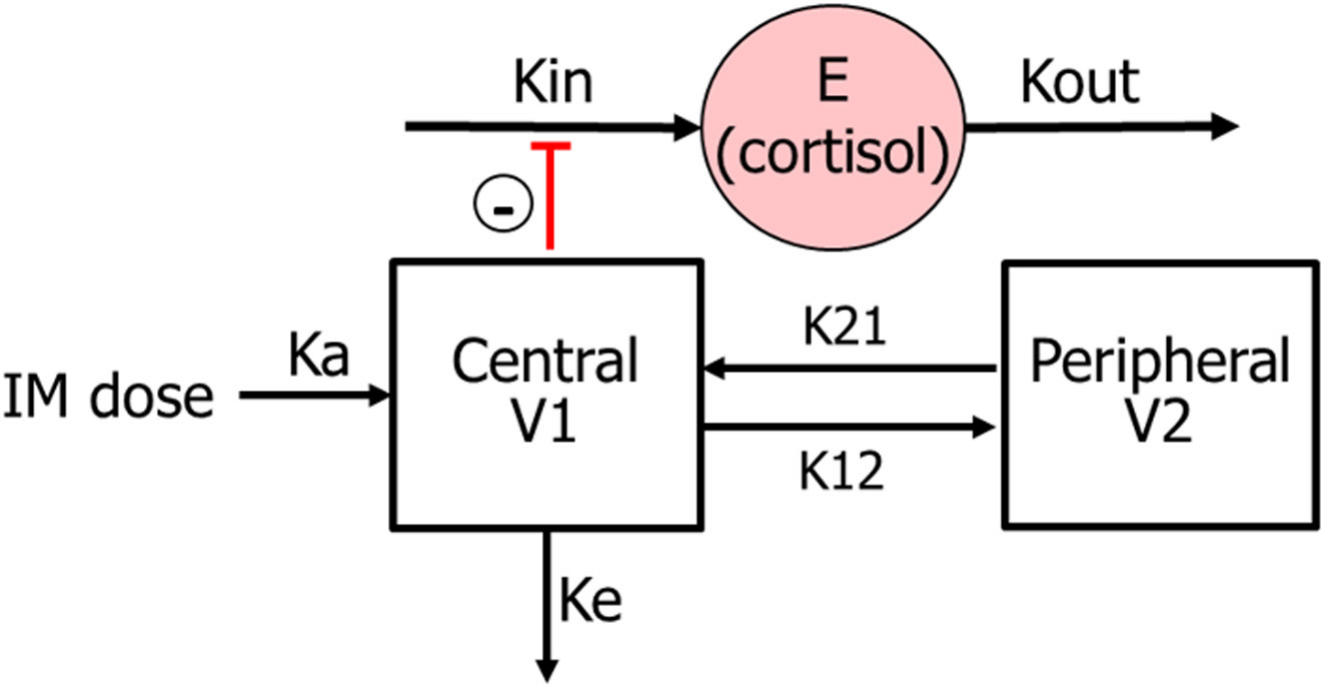
Schematic representation of the selected pharmacokinetic‐pharmacodynamic model for flunixin‐cortisol. Following intramuscular administration, flunixin is absorbed into the central compartment with a first‐order absorption rate constant ka, with drug movement between the central and peripheral compartments (rate constants K12 and K21) eliminated with elimination constant, Ke. In the absence of the drug, elevated cortisol concentration results from the balance of cortisol production (with zero‐order rate constant kin) and cortisol removal (with first‐order rate constant Kout). Flunixin induces an indirect reduction in cortisol (reduction of kin)

The final model used for the pop‐PK analysis of flunixin (see Figure 3) assumed first‐order kinetics of flunixin absorption following intramuscular administration, two‐compartmental disposition, and micro constant parameterization. Similar to the S‐(+)‐ketoprofen pop‐PK model, inter‐individual variability was expressed using an exponential model according to Equation (1). A multiplicative model was used to describe the residual random variability (*ε*) of the data for the plasma concentrations according to Equation (2).

### 2.7 The pharmacodynamic models

Raw ISF PGE2 and plasma cortisol concentrations for each of the NSAID‐treated groups were expressed as a percent difference from the control group (piglets that were castrated and tail docked without analgesia) before modeling. The pop‐PK/PD relationships were described using indirect PD response models, in which the effect (E) represents the percent difference in PGE2 or cortisol concentrations compared to the control group. Several alternative PD models (e.g., indirect response models, with or without the addition of a shape factor, with or without an effect compartment, and combination of these factors) were tested and discarded due to inferior performance before selection of the final PD models.

### 2.8 Ketoprofen‐PGE2 model

For the S‐(+)‐ketoprofen‐PGE2 model, it was assumed that the pharmacological effect (reduction of PGE2) is related to the ISF S‐(+)‐ketoprofen concentration (see Figure 1), and an indirect response PD model with inhibition of Kin was applied to link the S‐(+)‐ketoprofen concentration and anti‐inflammatory effect:

(3) $$\frac{\mathrm{dCe}}{\mathrm{dt}}=\mathrm{Ke}0\text{in}*C-\mathrm{Ke}0\text{out}*\mathrm{Ce}$$

(4) $$\frac{\mathrm{dE}}{\mathrm{dt}}=\mathrm{Kin}*\left(1-\frac{\text{Imax}*{\mathrm{Ce}}^{\gamma }}{{\mathrm{Ce}}^{\gamma }+\mathrm{IC}50^{\gamma }}\right)-\text{Kout}*E$$

where dCe/dt is the rate of change in S‐(+)‐ketoprofen ISF concentration, Ke0in and Ke0out are rate constants describing drug movement between the central and ISF compartments, dE/dt is the rate of change in PGE2 concentration, Ce is the S‐(+)‐ketoprofen concentration in ISF, γ is a curve‐fitting parameter, Imax is the maximal anti‐inflammatory effect, IC50 is the concentration that leads to 50% of the maximal inhibition of PGE2 production, Kin is a zero‐order constant for basal PGE2 production, and Kout is a first‐order rate constant for the removal of PGE2 from ISF. A multiplicative model was used to describe the residual random variability (ε) of the data for both ISF S‐(+)‐ketoprofen and PGE2 concentrations, where ε is the residual variability with a mean of zero and a variance of σ2, according to the equations:

(5) $$\text{CeObs}=\text{Cepred}*\left(1+\varepsilon \right)$$

(6) $$\text{EObs}=\text{Epred}*\left(1+\varepsilon \right)$$

where *CeObs* is the observed ISF concentration for the individual, and *Cepred* is the model predicted ISF concentration, *EObs* is the observed *PGE2* concentration for the individual, and Epred is the predicted *PGE2* concentration. Inter‐individual variability (variance of a parameter among different subjects or random effects) was expressed using an exponential model according to Equation (1).

### 2.9 Ketoprofen‐Cortisol model

It was assumed that the pharmacological effect (reduction of cortisol) is indirectly related to the S‐(+)‐ketoprofen concentration in plasma (see Figure 2) via a hypothetical effect compartment, and a sigmoidal indirect response PD model with inhibition of Kin was applied to link the NSAID concentration and cortisol as in Equation (4), where dE/dt is the rate of change in cortisol concentration, Ce is the NSAID concentration in the effect compartment, γ is a curve‐fitting parameter, Imax is the maximal inhibition of cortisol production, IC50 is the concentration that leads to 50% of the maximal inhibition of cortisol production, Kin is a zero‐order constant for basal cortisol production, and Kout is a first‐order rate constant for the removal of cortisol from plasma. A multiplicative model was used to describe the residual random variability (ε) of the data for cortisol concentrations, according to Equation (6), where EObs is the observed cortisol concentration for the individual and Epred is the model predicted cortisol concentration. Inter‐individual variability (variance of a parameter among different subjects or random effects) was expressed using an exponential model according to Equation (1).

### 2.10 Flunixin‐Cortisol model

It was assumed that the pharmacological effect (reduction of cortisol) is indirectly related to the flunixin concentration in the plasma (see Figure 3), and an indirect response PD model with inhibition of Kin was applied to link the flunixin concentration and cortisol:

(7) $$\frac{\mathrm{dE}}{\mathrm{dt}}=\mathrm{Kin}*\left(1-\frac{\text{Imax}*C}{C+\mathrm{IC}50}\right)-\text{Kout}*E$$

where dE/dt is the rate of change in cortisol concentration, C is the flunixin concentration in plasma, Imax is the maximal inhibition of cortisol production, IC50 is the concentration that leads to 50% of the maximal inhibition of cortisol production, Kin is a zero‐order constant for basal cortisol production, and Kout is a first‐order rate constant for the removal of cortisol from plasma. A Poisson model was used to describe the residual random variability (ε) of the data for cortisol concentrations:

(8) $$\text{EObs}=\text{Epred}+\left({\text{Epred}}^{0.5}*\varepsilon \right)$$

where EObs is the observed percent difference in cortisol concentration for the individual and Epred is the model predicted percent difference cortisol concentration. Inter‐individual variability (variance of a parameter among different subjects or random effects) was expressed using an exponential model according to Equation (1).

### 2.11 Median effective dose calculation

Using the finalized pop‐PK/PD models, the predicted time course of PGE2 and cortisol was simulated after single intramuscular doses of each NSAID at 15 doses ranging from 0 to 100 mg/kg via Monte Carlo simulations. The area under the time–response curve (AUR) was then calculated at each simulated dose, and the net effect was determined by subtracting the AUR corresponding to an absence of NSAID (i.e., a simulated dose of 0 mg/kg). The average drug response was then expressed as a percentage of the maximum possible average drug response, that is, the response obtainable for a very high dose of NSAID (e.g., 100 mg/kg). The maximum possible drug response percentage was plotted against the simulated dose and used to interpolate the median effective dose (ED50; GraphPad Prism version 9.1.2, GraphPad Software, San Diego, California, USA).

## 3 RESULTS

### 3.1 Ketoprofen‐PGE2

**FIGURE 4:**
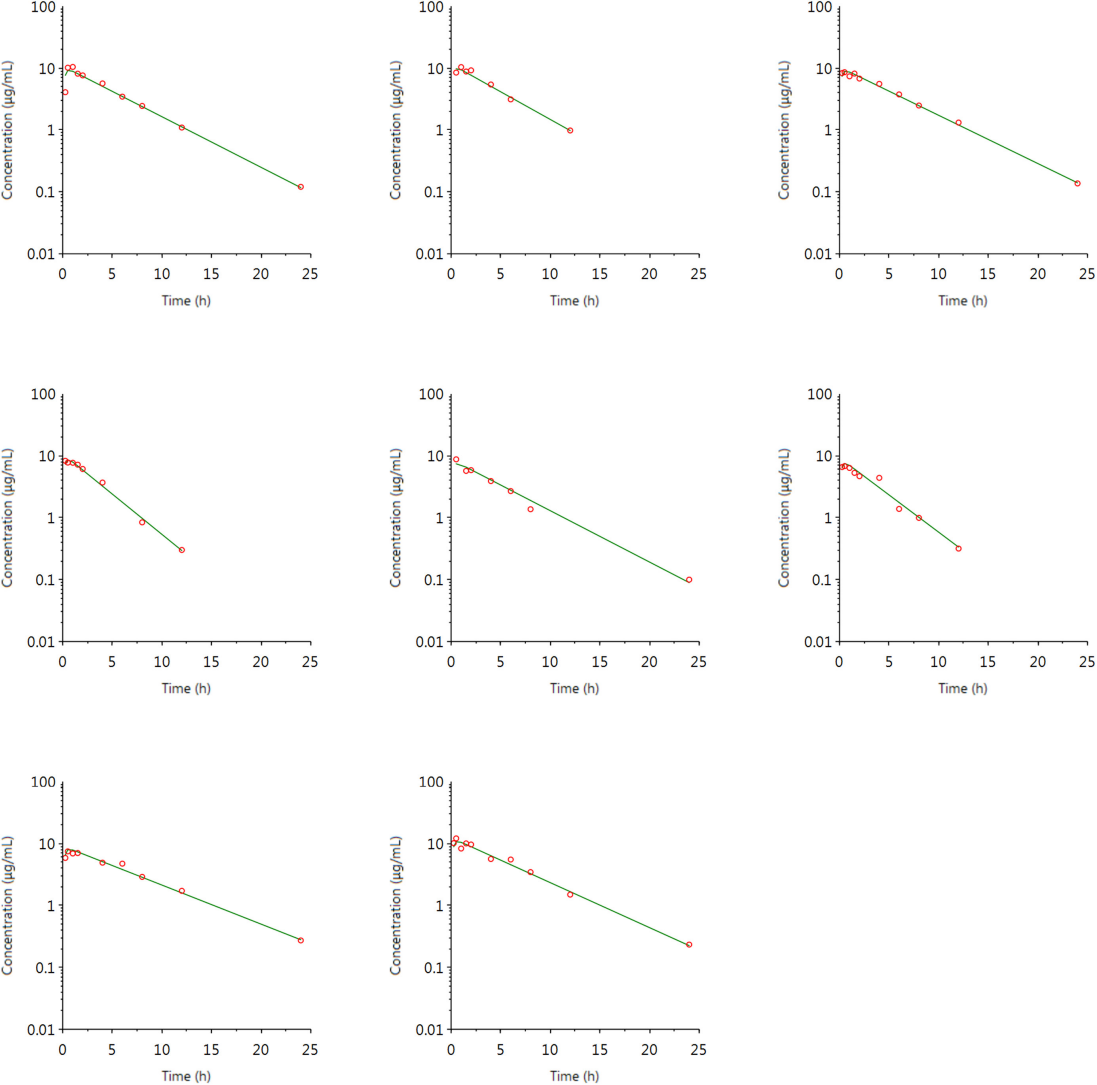
Individual fits of plasma concentrations for S‐(+)‐ketoprofen in piglets following an intramuscular dose of 3.0 mg/kg. The observed individual plasma concentrations are represented by the open circles, and the individual predictions (IPRED) are shown by the solid lines

**FIGURE 5:**
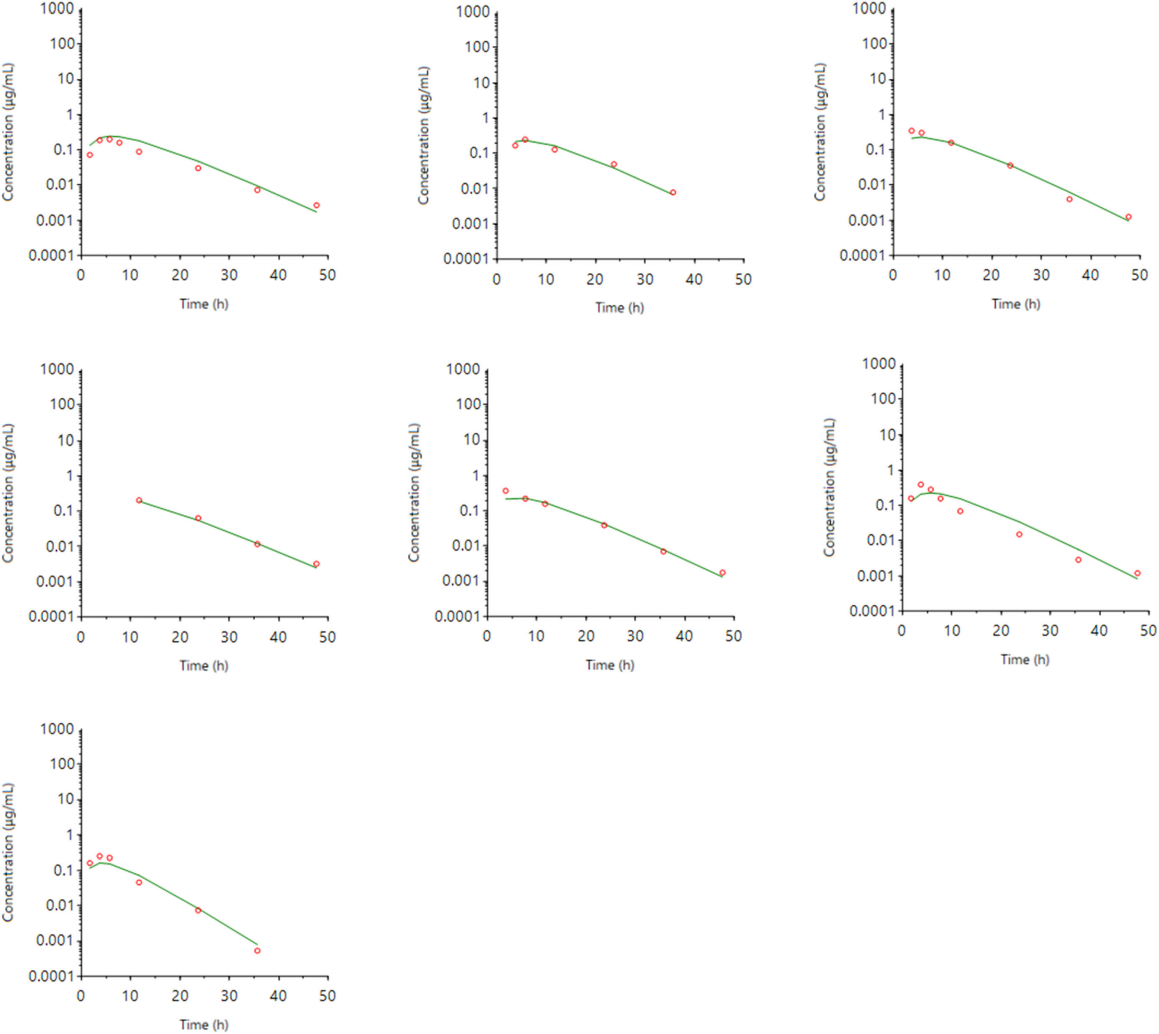
Individual fits of ISF concentrations for S‐(+)‐ketoprofen in piglets following an intramuscular dose of 3.0 mg/kg. The observed individual ISF concentrations are represented by the open circles, and the individual predictions (IPRED) are shown by the solid lines. Not shown for one individual due to missing data

**FIGURE 6:**
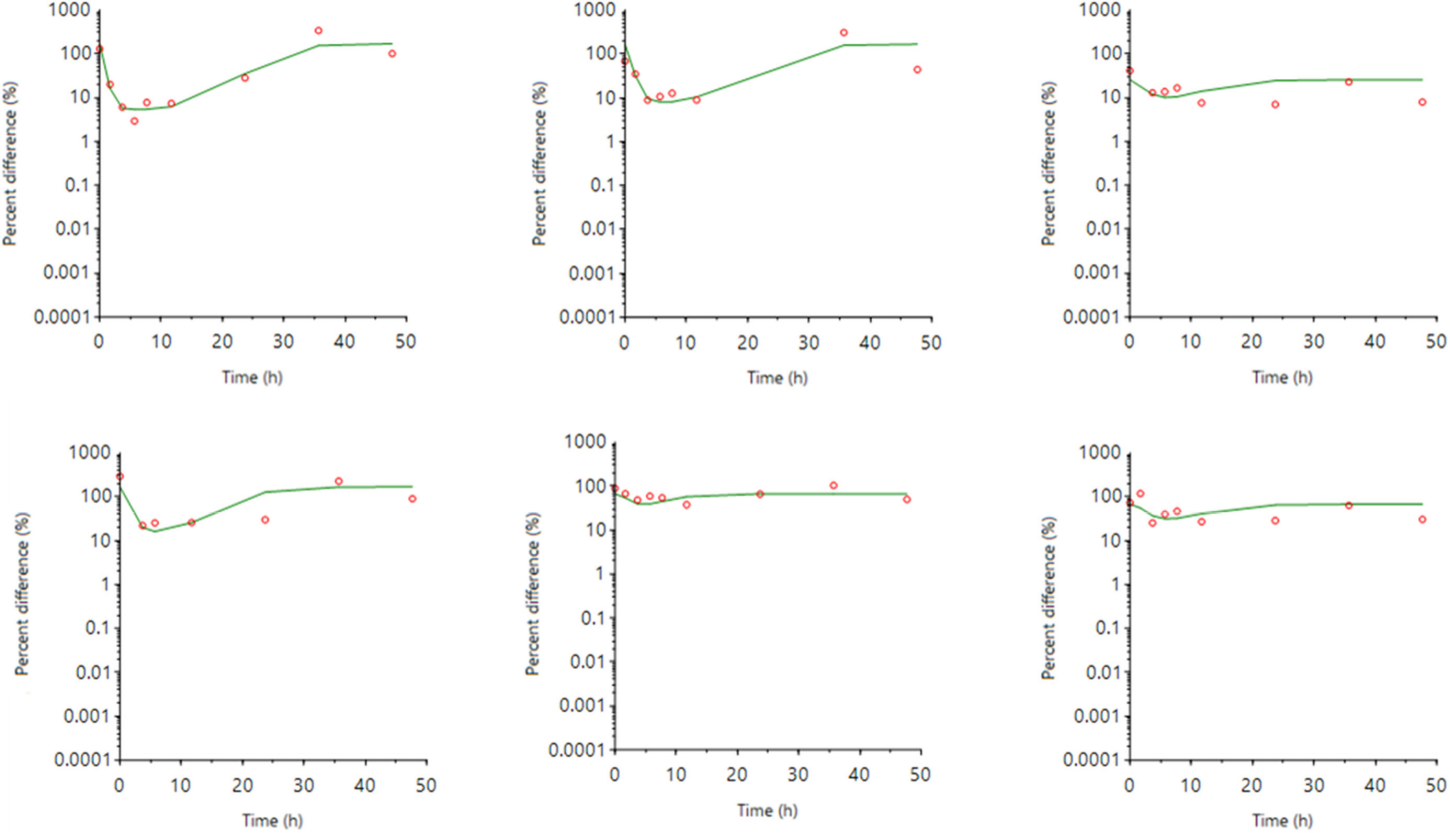
Individual fits of the percent difference in PGE2 concentrations in piglet ISF, compared with control piglets not given an analgesic, following an intramuscular dose of 3.0 mg/kg and castration and tail docking. The observed values are represented by the open circles, and the individual predictions (IPRED) are shown by the solid lines. Not shown for two individuals due to missing data

**FIGURE 7:**
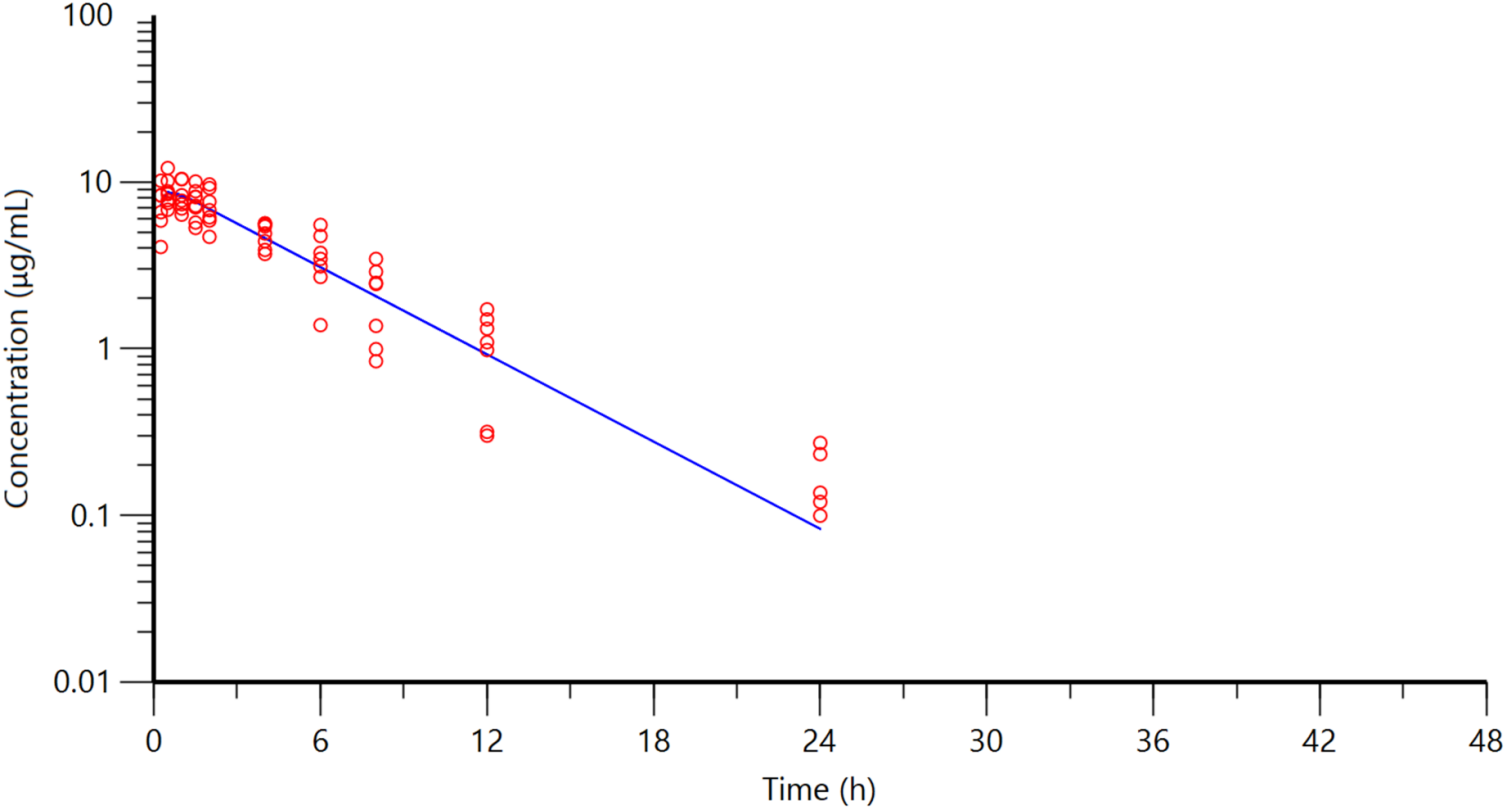
Plasma concentrations for S‐(+)‐ketoprofen in piglets following an intramuscular dose of 3.0 mg/kg. The observed individual ISF concentrations are represented by the open circles, and the population predictions (PRED) are shown by the solid lines

**FIGURE 8:**
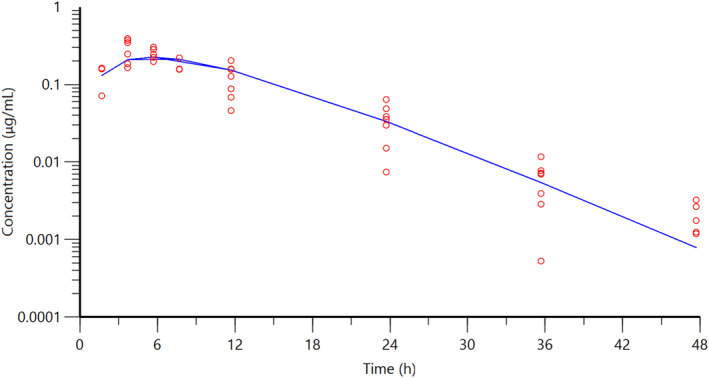
ISF concentrations for S‐(+)‐ketoprofen in piglets following an intramuscular dose of 3.0 mg/kg. The observed individual ISF concentrations are represented by the open circles, and the population predictions (PRED) are shown by the solid lines

**FIGURE 9:**
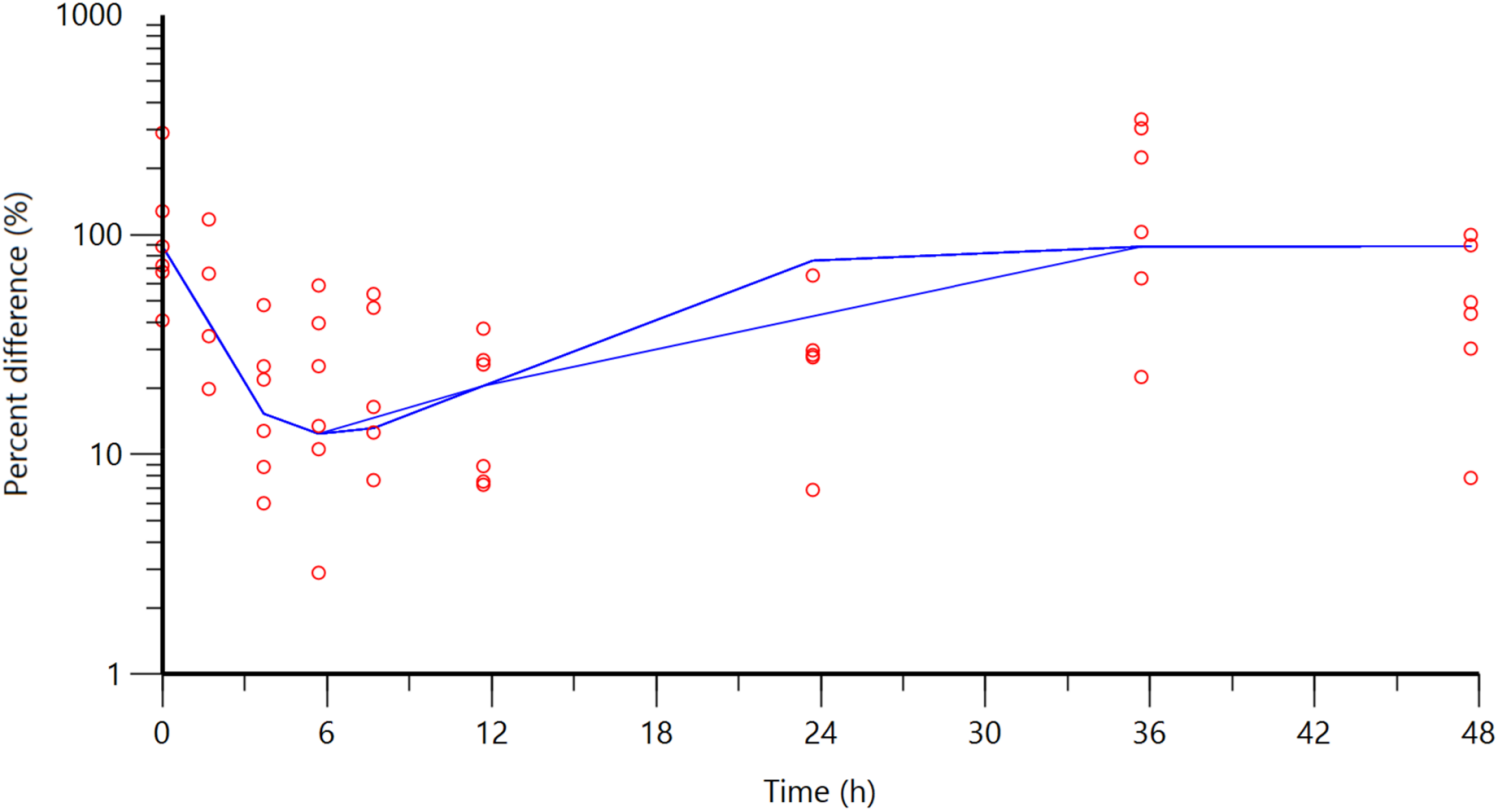
The percent difference in PGE2 concentrations in piglet ISF, compared to control piglets not given an analgesic, following an intramuscular dose of 3.0 mg/kg and castration and tail docking. The observed values are represented by the open circles, and the population predictions (PRED) are shown by the solid lines

**FIGURE 10:**
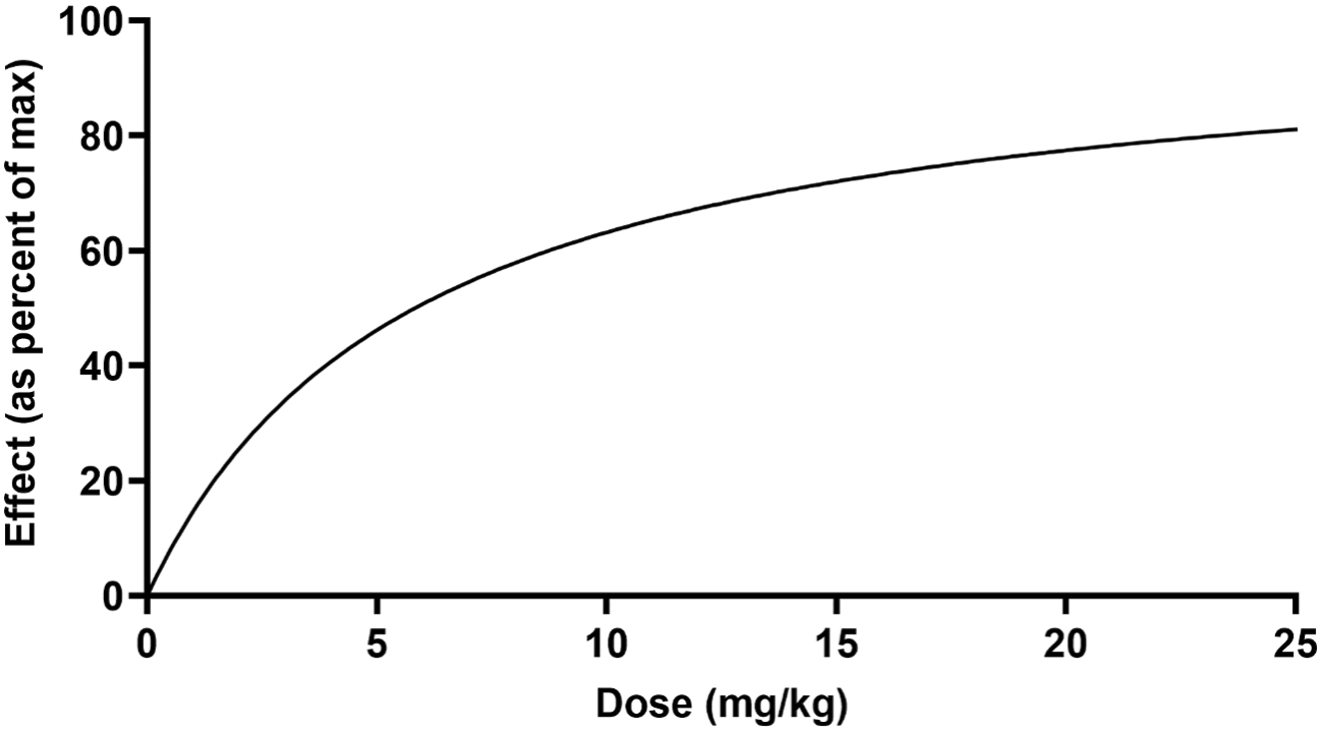
Relationship between ketoprofen administered intramuscularly and the inhibition of PGE2 production in piglets undergoing castration and tail‐docking over 48 h

The individual plasma concentration–time curves for S‐(+)‐ketoprofen are shown in Figure 4, and the individual interstitial fluid concentration–time curves are shown in Figure 5. The plasma and ISF PK parameters are presented in Tables 2 and 3. The absorption half‐life was short, and peak S‐(+)‐ketoprofen plasma concentration was reached rapidly. The elimination half‐life was 3.45 h. Penetration to the ISF (the ratio of AUC values of ISF and plasma) was only 6.64% of the plasma concentration. Figure 6 shows the individual percent difference in PGE2 concentrations over time compared to piglets that were castrated but not treated with an NSAID. The population fits for plasma S‐(+)‐ketoprofen, ISF S‐(+)‐ketoprofen, and ISF PGE2 are shown in Figures 7, 8, 9. The PD parameters are presented in Table 4. Pop‐PK/PD modeling of PGE2 gave an ISF IC50 of 0.08 μg/ml (estimated 1.2 μg/ml in plasma) for S‐(+)‐ketoprofen and an ED50 of 5.83 mg/kg. Simulation of the dose–effect relationship showed that a dose of 3.0 mg/kg corresponds to a drug response of 33.97% of the maximal possible response (Figure 10). The visual predictive checks and the simulations of the effect following 15 different doses (0–100 mg/kg) are provided in the Figures (S1‐S4).

**TABLE 2:** Population‐pharmacokinetic estimates for S‐(+)‐ketoprofen in piglet plasma, following administration to piglets intramuscularly at a dose of 3.0 mg/kg before castration and tail‐docking

Parameter	Estimate (CI)	Units	CV%	IIV%	Bootstrap median estimate (CI)
Tmax	0.61 (0.49–0.74)	h	10.12	–	–
Cmax	8.78 (7.87–9.68)	μg/ml	5.16	–	–
Ka	5.63 (4.09–7.16)	1/h	13.65	–	5.55 (4.17–7.69)
Ka t_1/2_	0.12 (0.09–0.16)	h	13.65	–	–
Ke	0.20 (0.17–0.23)	1/h	8.43	23.31	0.20 (0.17–0.24)
Ke t1/2	3.45 (2.87–4.03)	h	7.64	–	–
MRT	4.98 (4.14–5.82)	h	8.43	–	–
AUC_0‐48h_	49.46 (32.22–68.85)	h.μg/ml	9.60	–	–
Vd/F	302.11 (270.43–333.79)	ml/kg	5.25	12.92	301.88 (275.39–335.61)
Cl/F	60.66 (49.05–72.32)	ml/h/kg	9.60	–	–

*Notes*: CI, 2.5% to 97.5% confidence interval; IIV, inter‐individual variability. Primary parameters: Ka, absorption rate constant; Ke, elimination rate constant; Vd/F, apparent volume of distribution (per fraction absorbed). Secondary parameters: Tmax, time of maximal concentration; Cmax, maximal concentration; Ka t1/2, absorption half‐life; Ke t1/2, elimination half‐life; MRT, mean residence time; AUC_0‐48h_, area under the concentration vs. time curve from 0–48 h; Cl/F, apparent total body clearance (per fraction absorbed). – Not applicable.

**TABLE 3:** Population‐pharmacokinetic estimates for S‐(+)‐ketoprofen in piglet interstitial fluid, following administration to piglets intramuscularly at a dose of 3.0 mg/kg before castration and tail‐docking

Parameter	Estimate (CI)	Units	CV%	IIV%	Bootstrap median estimate (CI)
Ke0in	0.012 (0.010–0.013)	1/h	8.36	8.63	0.012 (0.008–0.014)
Ke0in t1/2	59.91 (49.95–69.88)	h	8.36	–	–
Ke0out	0.17 (0.14–0.21)	1/h	10.57	9.22	0.17 (0.13–0.25)
Ke0out t1/2	3.98 (3.14–4.82)	h	10.57	–	–
AUC	3.58 (2.07–4.08)	h.μg/ml	20.95	–	–
Penetration factor	6.64 (5.40–9.85)	%	24.16	–	–

*Notes*: CI, 2.5% to 97.5% confidence interval; IIV, inter‐individual variability. Primary parameters: Ke0in and Ke0out, rate constants describing drug movement between the central and ISF compartments. Secondary parameters: Tmax, time of maximal concentration; Cmax, maximal concentration; AUC, area under the concentration vs. time curve from 0–48 h; Penetration factor, the ratio of AUC values of ISF and plasma. – Not applicable.

**TABLE 4:** Population pharmacodynamic estimates describing the inhibitory effect of S‐(+)‐ketoprofen on interstitial fluid PGE2 production, following administration to piglets intramuscularly at a dose of 3.0 mg/kg before castration and tail‐docking

Parameter	Estimate (CI)	Units	CV%	IIV%	Bootstrap median estimate (CI)
Kin	207.20 (57.70–356.69)	%/h	36.26	–	195.90 (4.63–372.03)
Kout	2.34 (1.22–3.46)	1/h	24.09	–	2.19 (0.09–2.85)
Imax	97.33 (94.41–100.25)	%	1.51	–	96.80 (90.11–130.49)
ISF IC50	0.08 (0.02–0.15)	μg/ml	39.37	6.39	0.07 (0.03–0.17)
Plasma IC50	1.2	μg/ml	–	–	–
Gamma	2.07 (1.10–3.05)		23.55	–	2.51 (1.20–4.95)
ED50	5.83	mg/kg	–	–	–

*Notes*: CI, 2.5% to 97.5% confidence interval; IIV, inter‐individual variability. Primary parameters: Kin, zero‐order constant for basal PGE2 production; Kout, first‐order rate constant for the removal of PGE2 from ISF; Imax, maximal anti‐inflammatory effect; ISF IC50, the concentration that leads to 50% of the maximal inhibition of PGE2 production in ISF; gamma, exponent expressing sigmoidicity of the concentration–effect relationship. Secondary parameters: Plasma IC50, estimated by ISF IC50 divided by the penetration factor; ED50, median effective dose over 48 h. – Not applicable.

### 3.2 Ketoprofen‐Cortisol

**FIGURE 11:**
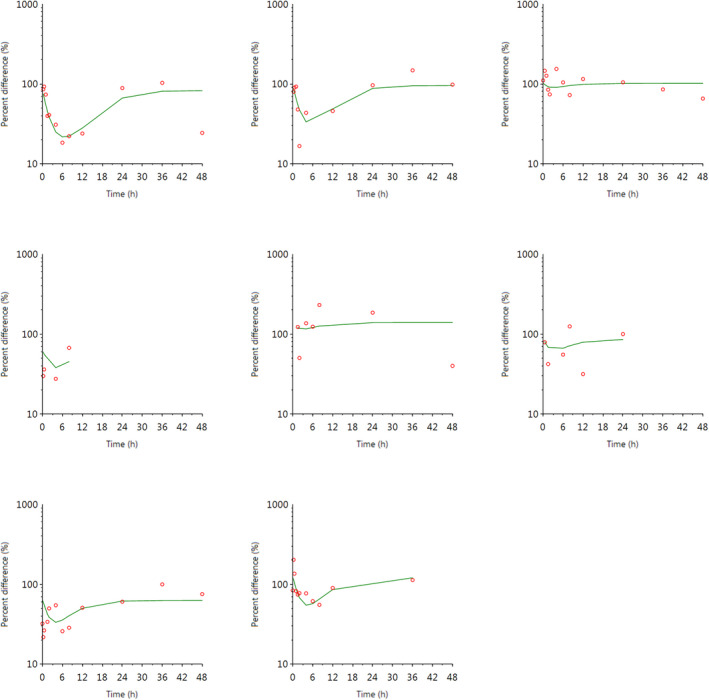
Individual fits of the percent difference in cortisol concentrations in piglet plasma, compared to control piglets not given an analgesic, following an intramuscular dose of 3.0 mg/kg and castration and tail docking. The observed values are represented by the open circles, and the individual predictions (IPRED) are shown by the solid lines

**FIGURE 12:**
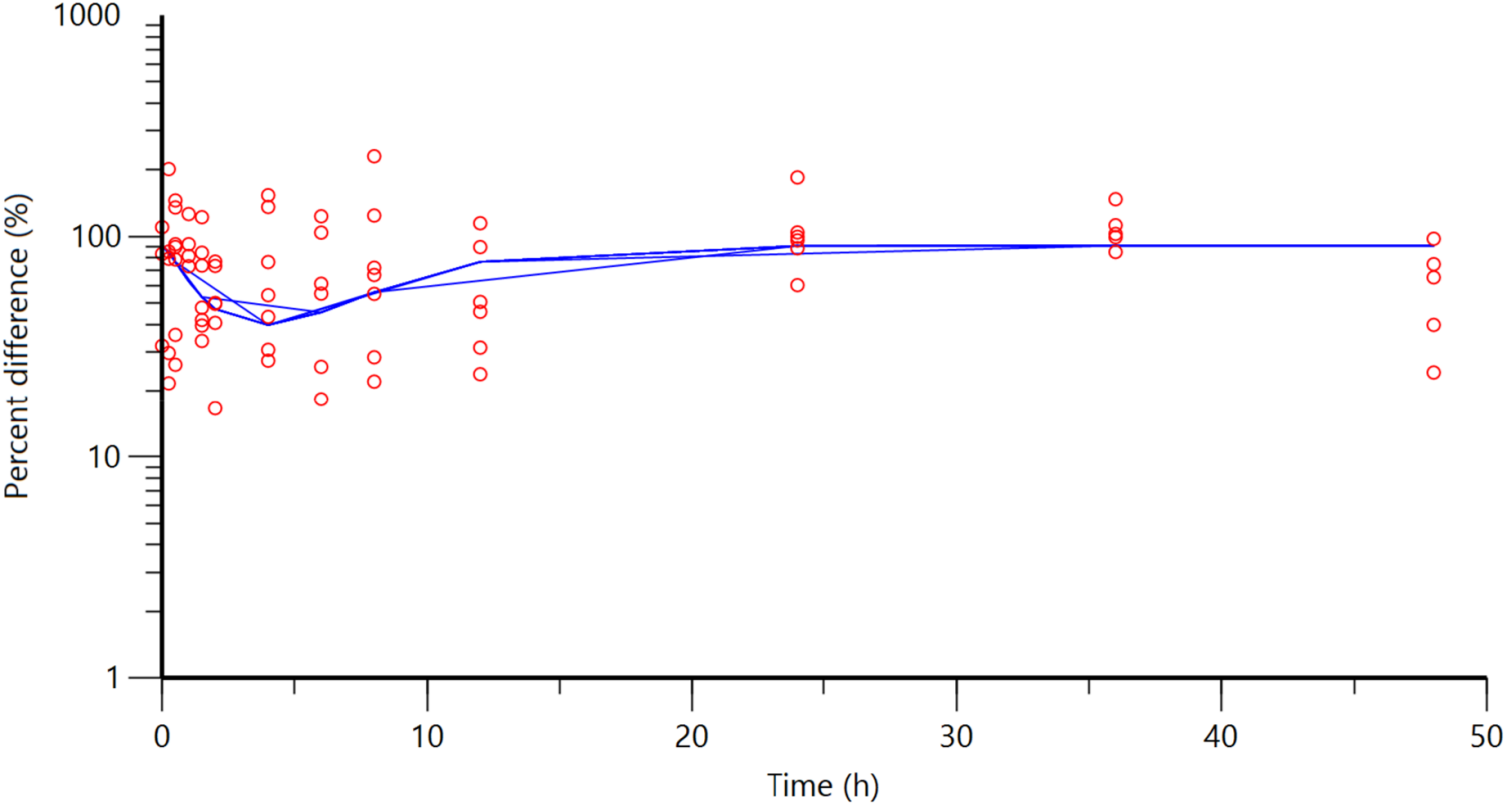
The percent difference in cortisol concentrations in piglet plasma, compared to control piglets not given an analgesic, following an intramuscular dose of 3.0 mg/kg and castration and tail docking. The observed values are represented by the open circles, and the population predictions (PRED) are shown by the solid lines

**FIGURE 13:**
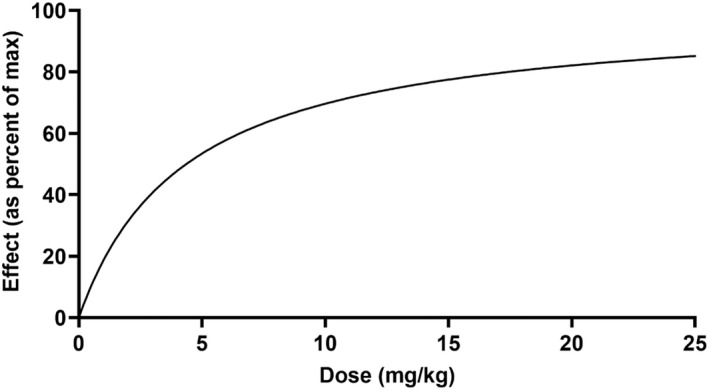
The relationship between ketoprofen administered intramuscularly and the inhibition of cortisol production in piglets undergoing castration and tail‐docking over 48 h

The results for the plasma pop‐PK model are shown in the section above. Figure 11 shows the individual percent difference in cortisol concentrations over time compared to piglets that were castrated but not treated with an NSAID. The population fits for cortisol are shown in Figure 12. The PD parameters are presented in Table 5. Pop‐PK/PD modeling of cortisol gave an IC50 of 2.56 μg/ml for S‐(+)‐ketoprofen and an ED50 of 4.36 mg/kg. Simulation of the dose–effect relationship showed that a dose of 3.0 mg/kg corresponds to a drug response of 40.75% of the maximal possible response (Figure 13). The visual predictive checks and the simulations of the effect following 15 different doses (0–100 mg/kg) are provided in the Figures (S5‐S6).

**TABLE 5:** Population pharmacodynamic estimates describing the inhibitory effect of S‐(+)‐ketoprofen on plasma cortisol production, following administration to piglets intramuscularly at a dose of 3.0 mg/kg prior to castration and tail‐docking

Parameter	Estimate (CI)	Units	CV%	IIV%	Bootstrap median estimate (CI)
Ke0	15.35 (0.60–30.10)	1/h	48.56	–	14.72 (1.06–185.88)
Kin	65.46 (29.04–101.87)	%/h	28.11	30.28	86.38 (44.06–1784.49)
Kout	0.72 (0.36–1.08)	1/h	25.41	–	0.87 (0.57–18.70)
Imax	73.00 (54.56–91.07)	%	12.67	–	66.00 (41.40–78.51)
IC50	2.56 (0.75–4.37)	μg/ml	35.68	124.08	2.44 (0.86–7.94)
Gamma	1.90 (0.78–3.03)		29.86	78.87	2.95 (1.48–29.81)
ED50	4.36	mg/kg	–	–	–

*Notes*: CI, 2.5% to 97.5% confidence interval; IIV, inter‐individual variability. Primary parameters: Kin, zero‐order constant for basal cortisol production; Kout, first‐order rate constant for the removal of cortisol; Imax, maximal anti‐inflammatory effect; IC50, the concentration that leads to 50% of the maximal inhibition of cortisol production; gamma, exponent expressing sigmoidicity of the concentration–effect relationship. Secondary parameters: ED50, median effective dose over 48 h. – Not applicable.

### 3.3 Flunixin‐Cortisol

**FIGURE 14:**
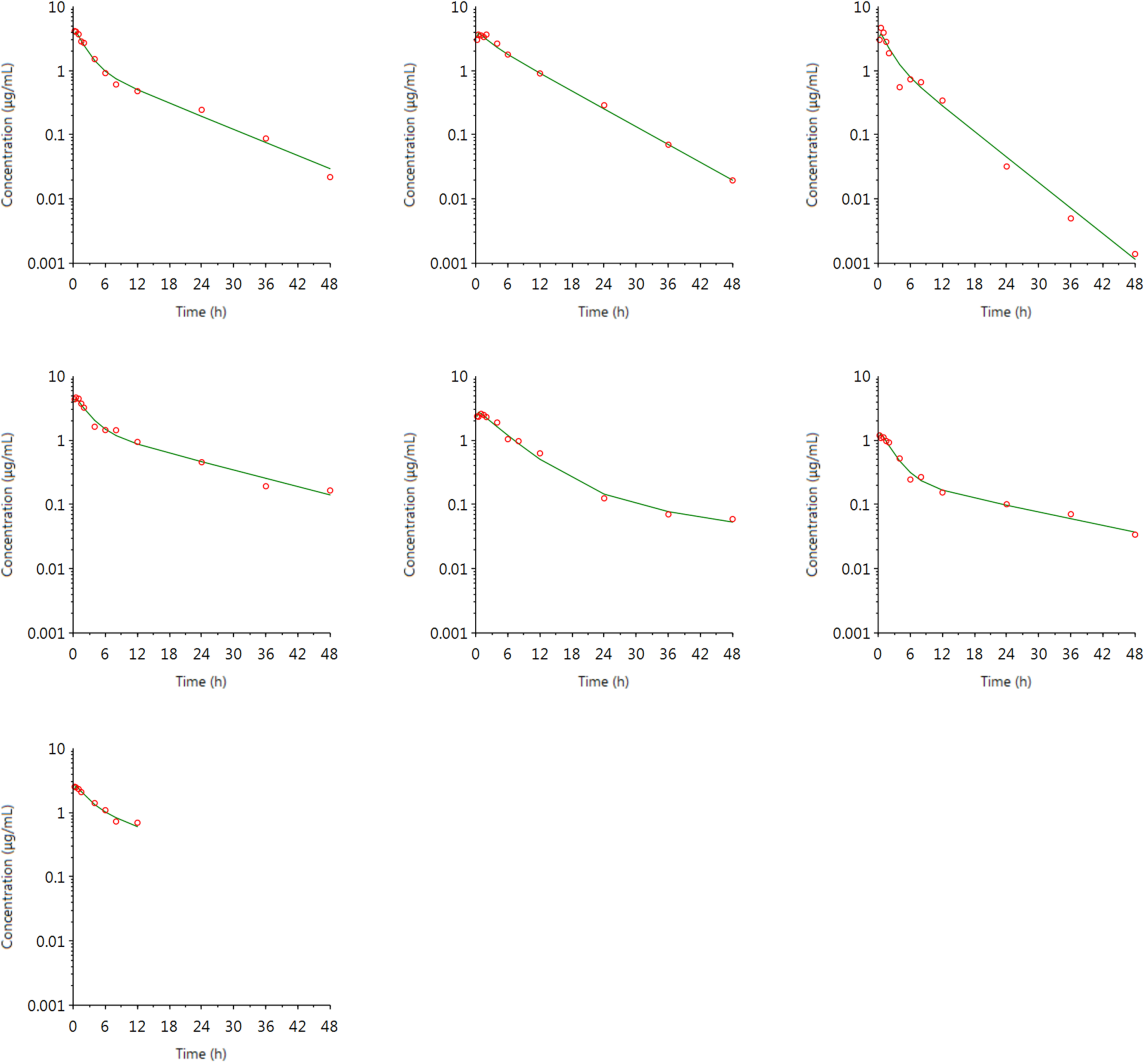
Individual fits of plasma concentrations for flunixin in piglets following an intramuscular dose of 2.2 mg/kg. The observed individual plasma concentrations are represented by the open circles, and the individual predictions (IPRED) are shown by the solid lines. Not shown for one individual due to missing data

**FIGURE 15:**
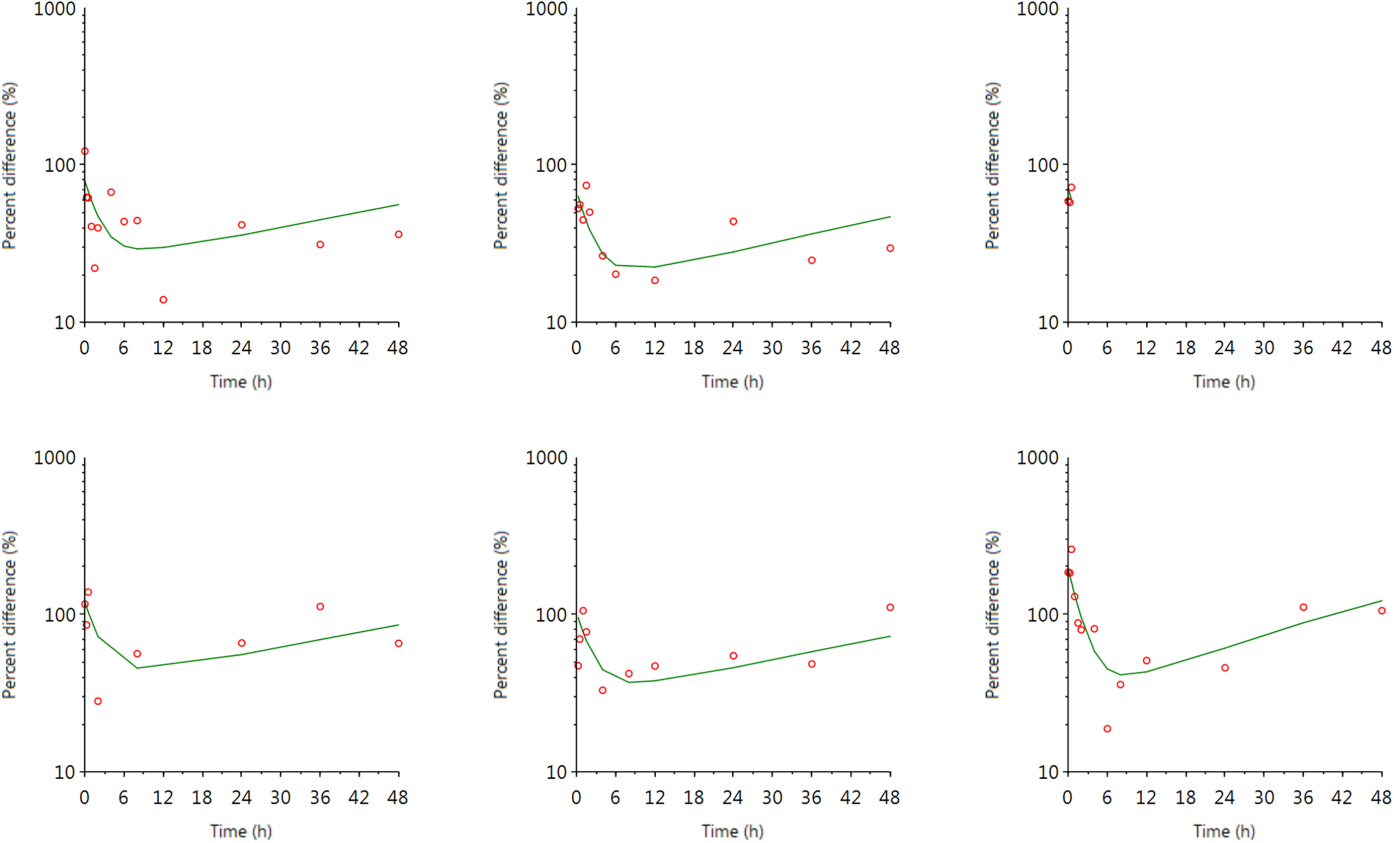
Individual fits of the percent difference in cortisol concentrations in piglet plasma, compared to control piglets not given an analgesic, following an intramuscular dose of 3.0 mg/kg and castration and tail docking. The observed values are represented by the open circles, and the individual predictions (IPRED) are shown by the solid lines. Not shown for two individuals due to missing data

**FIGURE 16:**
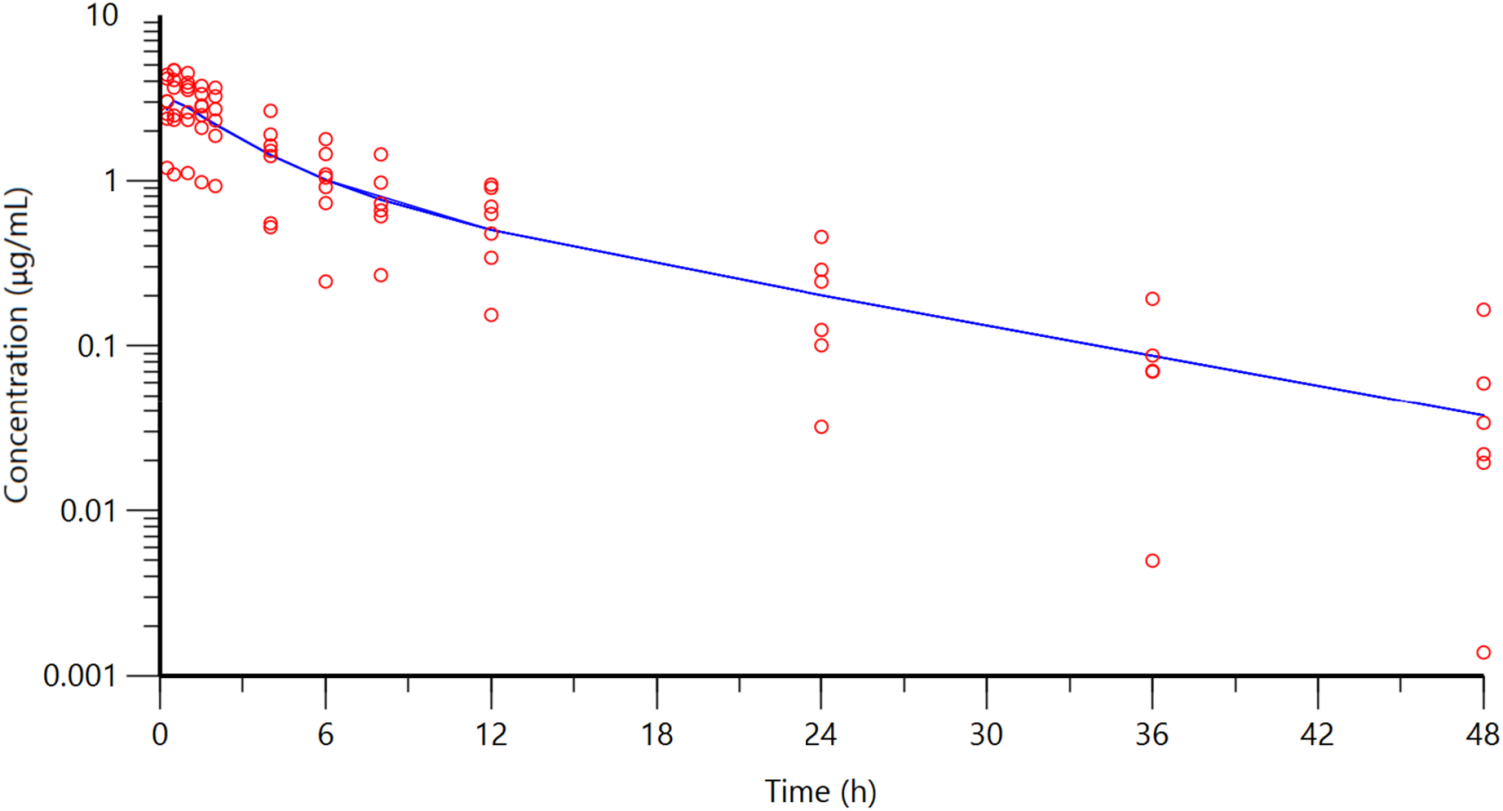
Plasma concentrations for flunixin in piglets following an intramuscular dose of 2.2 mg/kg. The observed individual ISF concentrations are represented by the open circles, and the population predictions (PRED) are shown by the solid lines

**FIGURE 17:**
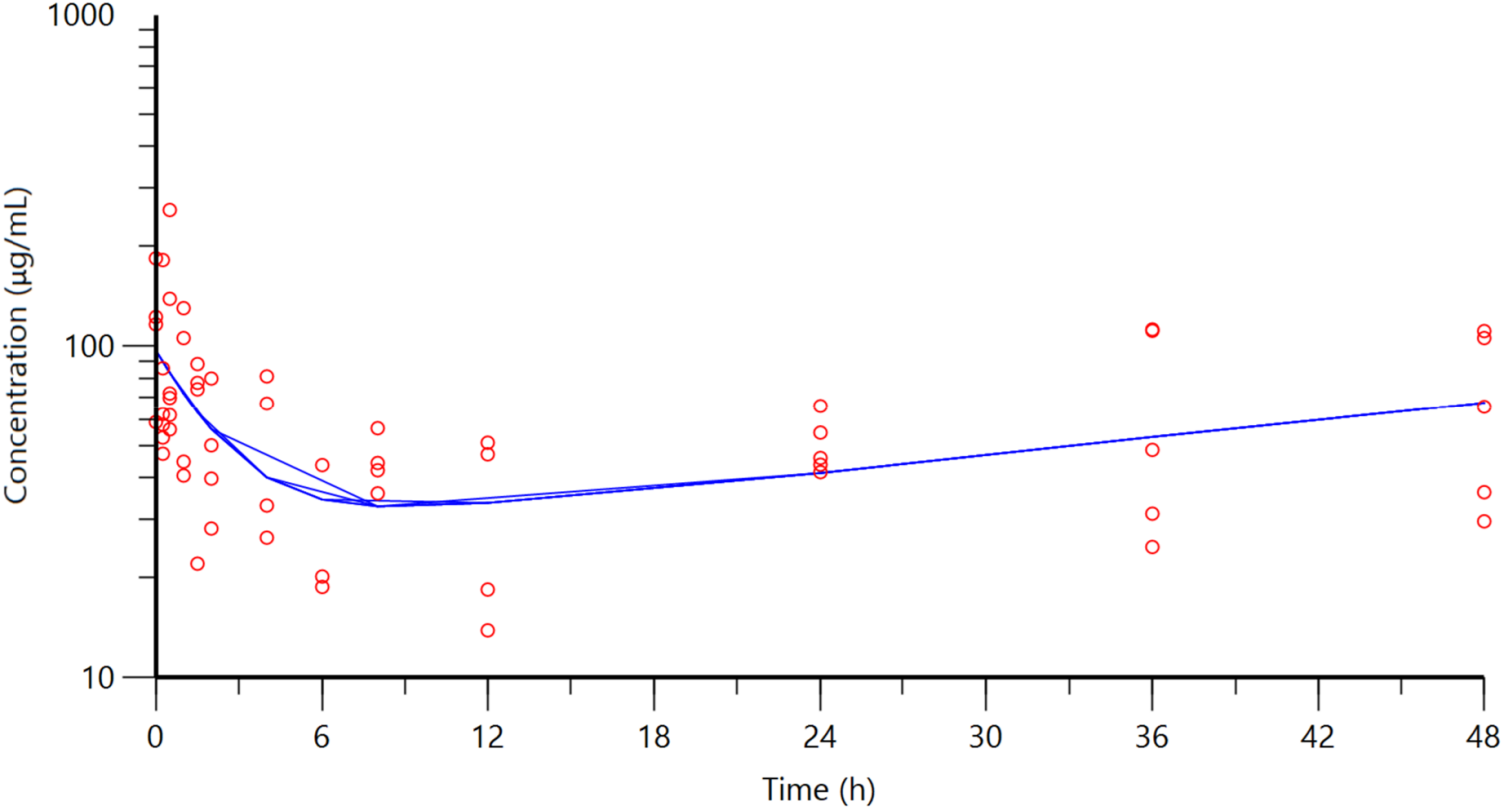
Percent difference in cortisol concentrations in piglet plasma, compared to control piglets not given an analgesic, following an intramuscular dose of 3.0 mg/kg and castration and tail docking. The observed values are represented by the open circles, and the population predictions (PRED) are shown by the solid lines

**FIGURE 18:**
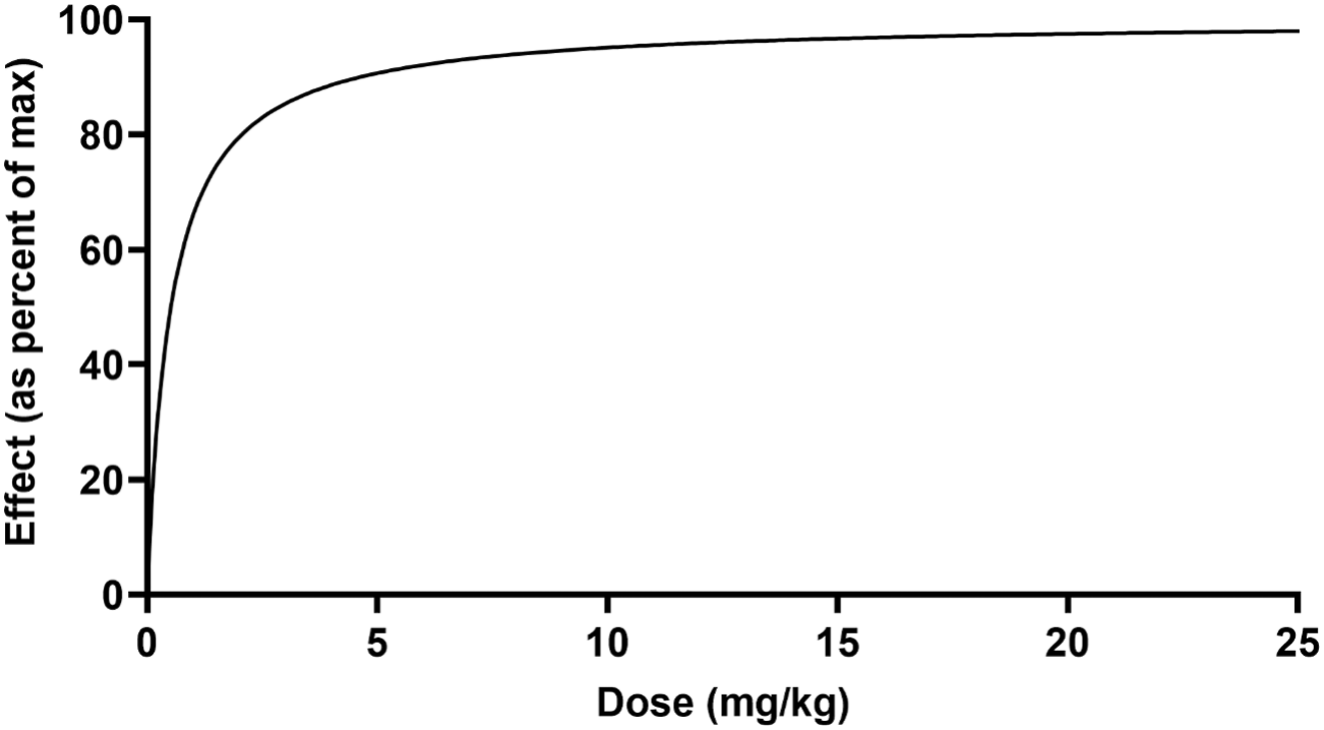
Relationship between flunixin administered intramuscularly and the inhibition of cortisol production in piglets undergoing castration and tail‐docking over 48 h

The individual plasma concentration–time curves for flunixin are shown in Figure 14, and the plasma pop‐PK parameters are presented in Table 6. The absorption half‐life was short, and peak flunixin plasma concentration was reached rapidly. The elimination half‐life was 4.42 h. Figure 15 shows the individual percent difference in cortisol concentrations over time compared to piglets that were castrated but not treated with an NSAID. The population fits for plasma flunixin and plasma cortisol are shown in Figures 16 and 17. The PD parameters are presented in Table 7. Pop‐PK/PD modeling of cortisol gave an IC50 of 0.06 μg/ml and an ED50 of 0.51 mg/kg for flunixin. Simulation of the dose–effect relationship showed that a dose of 2.2 mg/kg corresponds to a drug response of 81.05% of the maximal possible response (Figure 18). The visual predictive checks and the simulations of the effect following 15 different doses (0–100 mg/kg) are provided in the Figures (S7‐S9).

**TABLE 6:** Population‐pharmacokinetic estimates for flunixin in piglet plasma, following administration to piglets intramuscularly at a dose of 2.2 mg/kg before castration and tail‐docking

Parameter	Estimate (CI)	Units	CV%	IIV%	Bootstrap median estimate (CI)
Tmax	0.51 (0.36–0.66)	h	14.88	–	–
Cmax	3.03 (2.06–4.00)	μg/ml	16.01	–	–
Ka	6.68 (3.71–9.65)	1/h	22.29	–	6.66 (3.65–8.72)
Ka t_1/2_	0.10 (0.06–0.15)	h	22.29	–	–
Ke	0.16 (0.11–0.20)	1/h	13.60	31.44	0.16 (0.12–0.25)
Ke t1/2	4.42 (3.22–5.62)	h	13.60	–	–
K12	0.11 (0.02–0.02)	1/h	40.66	71.98	0.11 (0.03–0.42)
K21	0.16 (0.03–0.29)	1/h	41.66	94.78	0.18 (0.06–0.37)
MRT	6.38 (4.65–8.11)	h	13.60	–	–
AUC	22.06 (1.32–30.80)	h.μg/ml	19.86	–	–
Vd/F	636.17 (425.09–847.24)	ml/kg	16.63	41.07	618.87 (413.13–904.80)
Cl/F	99.73 (60.21–139.26)	ml/h/kg	19.86	–	–

*Notes*: CI, 2.5% to 97.5% confidence interval; IIV, inter‐individual variability. Primary parameters: Ka, absorption rate constant; Ke, elimination rate constant; Vd/F, apparent volume of distribution (per fraction absorbed). Secondary parameters: Tmax, time of maximal concentration; Cmax, maximal concentration; Ka t1/2, absorption half‐life; Ke t1/2, elimination half‐life; MRT, mean residence time; AUC, area under the concentration vs. time curve; Cl/F, apparent total body clearance (per fraction absorbed). – Not applicable.

**TABLE 7:** Population pharmacodynamic estimates describing the inhibitory effect of flunixin on plasma cortisol production, following administration to piglets intramuscularly at a dose of 2.2 mg/kg before castration and tail‐docking

Parameter	Estimate (CI)	Units	CV%	IIV%	Bootstrap median estimate (CI)
Kin	44.93 (14.35–75.51)	%/(h)	33.89	38.58	48.53 (22.20–148.40)
Kout	0.46 (0.21–0.71)	1/h	26.78	–	0.49 (0.29–1.35)
Imax	72.02 (55.83–88.22)	%	11.20	13.26	72.35 (54.57–87.00)
IC50	0.059 (0.002–0.116)	μg/ml	47.70	10.62	0.070 (0.001–0.283)
ED50	0.51	–	–	–	–

*Notes*: CI, 2.5% to 97.5% confidence interval; IIV, inter‐individual variability. Primary parameters: Kin, zero‐order constant for basal cortisol production; Kout, first‐order rate constant for the removal of cortisol; Imax, maximal anti‐inflammatory effect; IC50, the concentration that leads to 50% of the maximal inhibition of cortisol production. Secondary parameters: ED50, median effective dose over 48 h. – Not applicable.

## 4 DISCUSSION

This study is the first to report an IC50 value associated with PGE2 reduction for any NSAID in pigs and the first to report an IC50 value associated with reduction in cortisol for NSAIDs in any species. This study is also the first to assess the PK/PD relationships directly in piglets that are surgically castrated and tail docked, rather than using an induced inflammation model.

The S‐(+)‐enantiomers of 2‐arylpropionic acid derivatives, or “profens,” are much more potent inhibitors of COX than the R‐(−)‐enantiomers (Suesa et al., 1993), and the S‐(+)‐enantiomers have therefore been considered the pharmacologically active enantiomers (Cabré et al., 1998). Although R‐(−)‐ketoprofen has been reported to play a role in analgesia with likely a centrally mediated mechanism of action independent of COX‐2 inhibition, (Ghezzi et al., 1998) S‐(+)‐ketoprofen predominates over R‐(−)‐ketoprofen in terms of plasma exposure following intramuscular administration of racemic ketoprofen in piglets (Fosse, Horsberg, et al., 2011; Nixon et al., 2020) with rapid chiral conversion to S‐(+)‐ketoprofen. Therefore, the pop‐PK/PD modeling in this study was performed solely using S‐(+)‐enantiomer concentrations of ketoprofen.

The pharmacokinetic parameters Tmax, Cmax, and AUC of S‐(+)‐ketoprofen were similar to the previously reported noncompartmental analysis using this dataset (Nixon et al., 2020). The elimination half‐life of 3.45 h was also similar to the previously reported terminal half‐life of 3.50 h. These values are also comparable to values reported for similar age piglets by other studies (Fosse, Horsberg, et al., 2011; Fosse, Toutain, et al., 2011). The estimated IC50 of 1.2 μg/ml for S‐(+)‐ketoprofen using PGE2 as a biomarker was higher than that reported in other large animal species; calf 0.042 μg/ml, goat 0.003 μg/ml, horse 0.033 μg/ml, and sheep 0.007 μg/ml (Arifah et al., 2003; Landoni et al., 1999; Landoni & Lees, 1995b, 1996), although these historic EC50 values generated via basic indirect effect models are regarded as dose‐dependent variables rather than parameters (Dayneka et al., 1993) and care must be taken in comparing these values. PK/PD modeling of ketoprofen in piglets has been performed previously, however, that study utilized a kaolin‐induced inflammation model and mechanical nociceptive threshold (MNT) testing, an outcome with a different mechanism of action (Fosse, Toutain, et al., 2011). Based on MNTs, the estimated ED50 from that study was 2.5 mg/kg, which is lower than the estimates in the present study (5.83 mg/kg when using PGE2 as a biomarker, and 4.36 mg/kg when using cortisol as a biomarker). However, IC50 calculated when using cortisol as a biomarker was high, at 2.56 μg/ml. As prostaglandins stimulate ACTH and cortisol release (Sheil et al., 2020), the effect of NSAIDs on cortisol is more indirect, as it is further down the pathway in the mechanism of action. Therefore, cortisol may be less sensitive to small changes in NSAID concentration. There was also a significant degree of inter‐individual variability (124.08%) associated with the IC50 for cortisol following ketoprofen administration. There is also marked individual variability in response to anti‐inflammatories in other species (Giraudel et al., 2005; Levy, 1998).

The pharmacokinetic parameters for flunixin were slightly different from the previously reported noncompartmental analysis (NCA) using this dataset (Nixon et al., 2020). A 2‐compartment model enables the distribution and elimination phases to be accounted for separately, which likely caused these slight differences. In addition, the Tmax and Cmax are taken directly from the raw data in an NCA, whereas a compartmental analysis calculates these from the predicted model fits. Based on the compartmental model, the Tmax was reached more rapidly (0.51 h vs. 0.85 h), but still similar to the Tmax of 0.61 h from another study in mature swine (Pairis‐Garcia et al., 2013). The AUC was slightly lower than the previous NCA report, which likely overestimated this value (22.06 h.μg/ml vs. 27.25 h.μg/ml). The elimination half‐life of 4.42 h was lower than the NCA report of 7.93 h; however, it was much closer to previously reported in similar age piglets (4.82 and 5.15 h [Levionnois et al., 2018]) and lower than that of mature swine (7.93 h). The IC50 for flunixin utilizing cortisol as a biomarker was 0.06 μg/ml. There are no previous PK/PD models for NSAIDs utilizing cortisol as an outcome for comparison; however, this value does compare to values measured for various NSAIDs in other large animal species for inhibiting PGE2 production. PK/PD modeling of flunixin in piglets has been performed previously; however, that study utilized a kaolin‐induced inflammation model and mechanical nociceptive threshold (MNT) testing. Based on MNTs, the estimated ED50 from that study was 6.6 mg/kg (Levionnois et al., 2018), differing quite significantly from the ED50 of 0.51 mg/kg estimated in the present study. Given the different model used (induced inflammation vs. surgical castration and tail‐docking), different outcome used, and different route of administration of the drug (intravenous vs. intramuscular), it is difficult to make comparisons. The same difficulties would apply to the above discussion of ketoprofen as well. In the present study, simulation of the dose–effect relationship showed that a dose of 3.0 mg/kg corresponds to a drug response of 81.05% of the maximal possible response. However, given the discrepancy between ED50 values, it would be pertinent to perform further research to ensure the most optimal dose is given.

This study does have some limitations worth consideration. The models utilized data from surrogate markers of the analgesic response (biomarkers more closely related to the anti‐inflammatory and stress response), and therefore the actual analgesic response may be different. In addition, relatively few piglets (*n* = 8) were included, and litter/sow parity were not accounted for in these population analyses, so the inter‐individual variability of the true population may not be optimally represented. The visual predictive plots are shown in the supplementary figures; however, the 5th and 95th percentiles of observed data for such a small sample (*n* = 8) are not robust and attention should be focused on the predicting performation around the median. Finally, the piglets in these studies were individually housed, which could influence the outcomes examined in this study compared with piglets housed together with the sow. However, a controlled environment was required to maintain the intravenous catheters and subcutaneous interstitial fluid probes. Further research would need to be conducted on‐farm to determine whether the ED50 values still hold when more confounding factors are included.

The ultimate goal of the pop‐PK/PD modeling was to ensure that the optimal dose regimen is given to piglets for pain mitigation at castration and tail‐docking. This study shows that the currently marketed doses of ketoprofen (3.0 mg/kg) and flunixin (2.2 mg/kg) correspond to a drug response of 33.97% (ketoprofen‐PGE2), 40.75% (ketoprofen‐cortisol), and 81.05% (flunixin‐cortisol) of the maximal possible responses. Given this information, flunixin may be the best NSAID to use in mitigating castration and tail‐docking pain at the current label dose.

## AUTHOR CONTRIBUTIONS

EN, RB and KM planned the experiment and performed the experiment together. The data was then analysed by EN and JC. EN drafted the manuscript. All authors revised the manuscript and approved the final version of the manuscript.

## ANIMAL WELFARE AND ETHICS STATEMENT

The animal study was reviewed and approved by North Carolina State University Institutional Animal Care and Use Committee.[Correction added on 06 September 2022, after first online publication: The Animal Welfare and Ethics Statement was included in this current version.]

## CONFLICT OF INTEREST

The authors declared no conflict of interest.

## Supporting information


Figures S1‐S9

## Data Availability

The data that support the findings of this study are available from the corresponding author upon reasonable request.
