# Supplementary material for: Pharmacokinetic/pharmacodynamic modeling of ketoprofen and flunixin at piglet castration and tail‐docking
Source: J Vet Pharmacol Ther. 2022 Jul 14;45(5):450–66. doi: 10.1111/jvp.13083 (PMC9541024; doi:10.1111/jvp.13083)
Supplement: Supplementary file 1 — Figures S1‐S9 [file JVP-45-450-s001.docx]

**Supplementary figures**


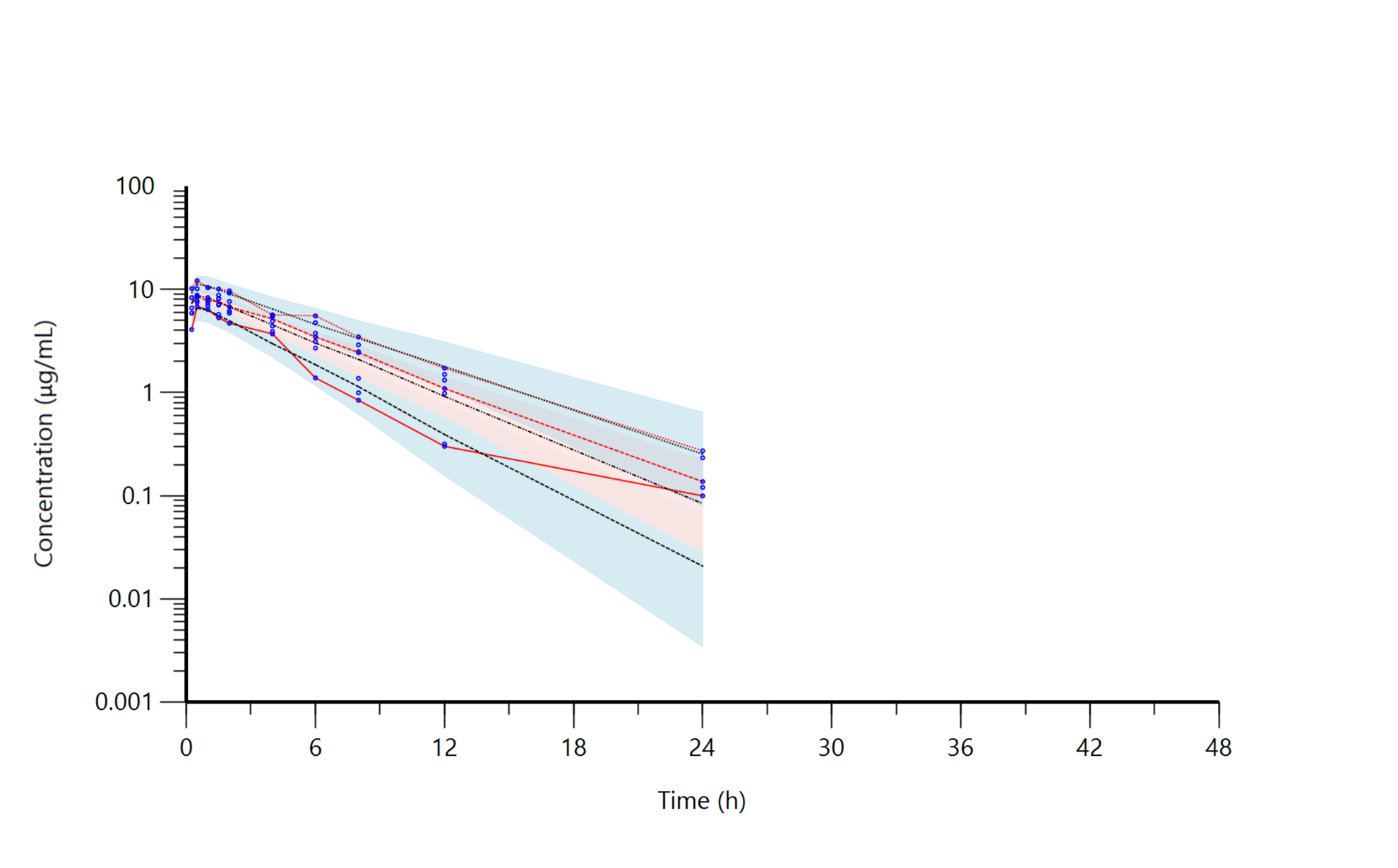


**Figure S1.** Visual predictive check (VPC) of the final model for ketoprofen in plasma (using 300 replicates). Observed plasma concentrations are depicted by the open circles. The 5th, 50th and 95th percentiles of the observed concentrations are represented by the red lines. The 5th, 50th and 95th percentiles of the predicted concentrations are represented by the black dashed lines. The 95% confidence intervals (CI) for the predicted 5th and 95th percentiles are represented by the blue shaded regions. The 95% CI for the predicted 50th percentile is represented by the red shaded region.


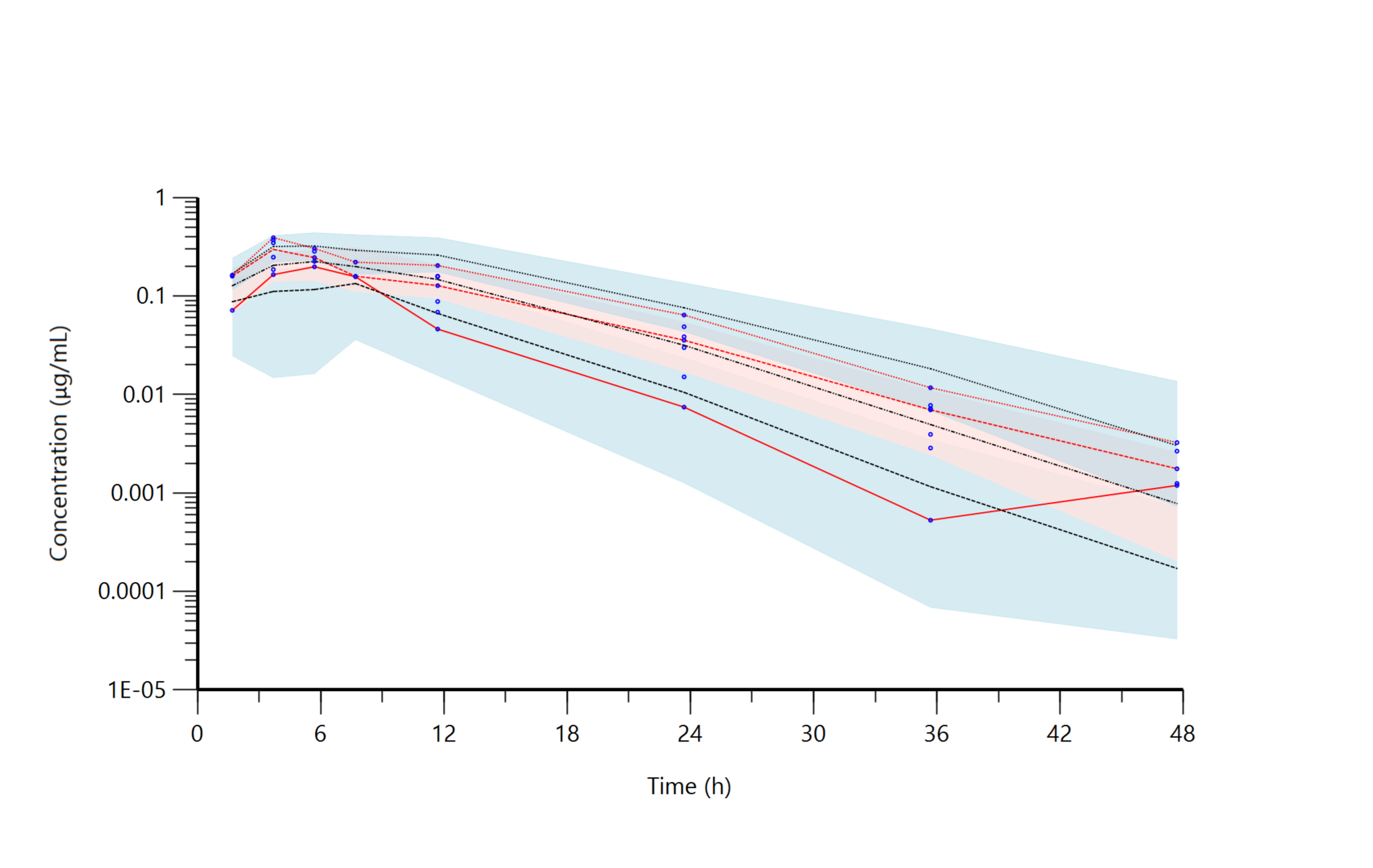


**Figure S2.** Visual predictive check (VPC) of the final model for ketoprofen in interstitial fluid (using 300 replicates). Observed interstitial fluid concentrations are depicted by the open circles. The 5th, 50th and 95th percentiles of the observed concentrations are represented by the red lines. The 5th, 50th and 95th percentiles of the predicted concentrations are represented by the black dashed lines. The 95% confidence intervals (CI) for the predicted 5th and 95th percentiles are represented by the blue shaded regions. The 95% CI for the predicted 50th percentile is represented by the red shaded region.


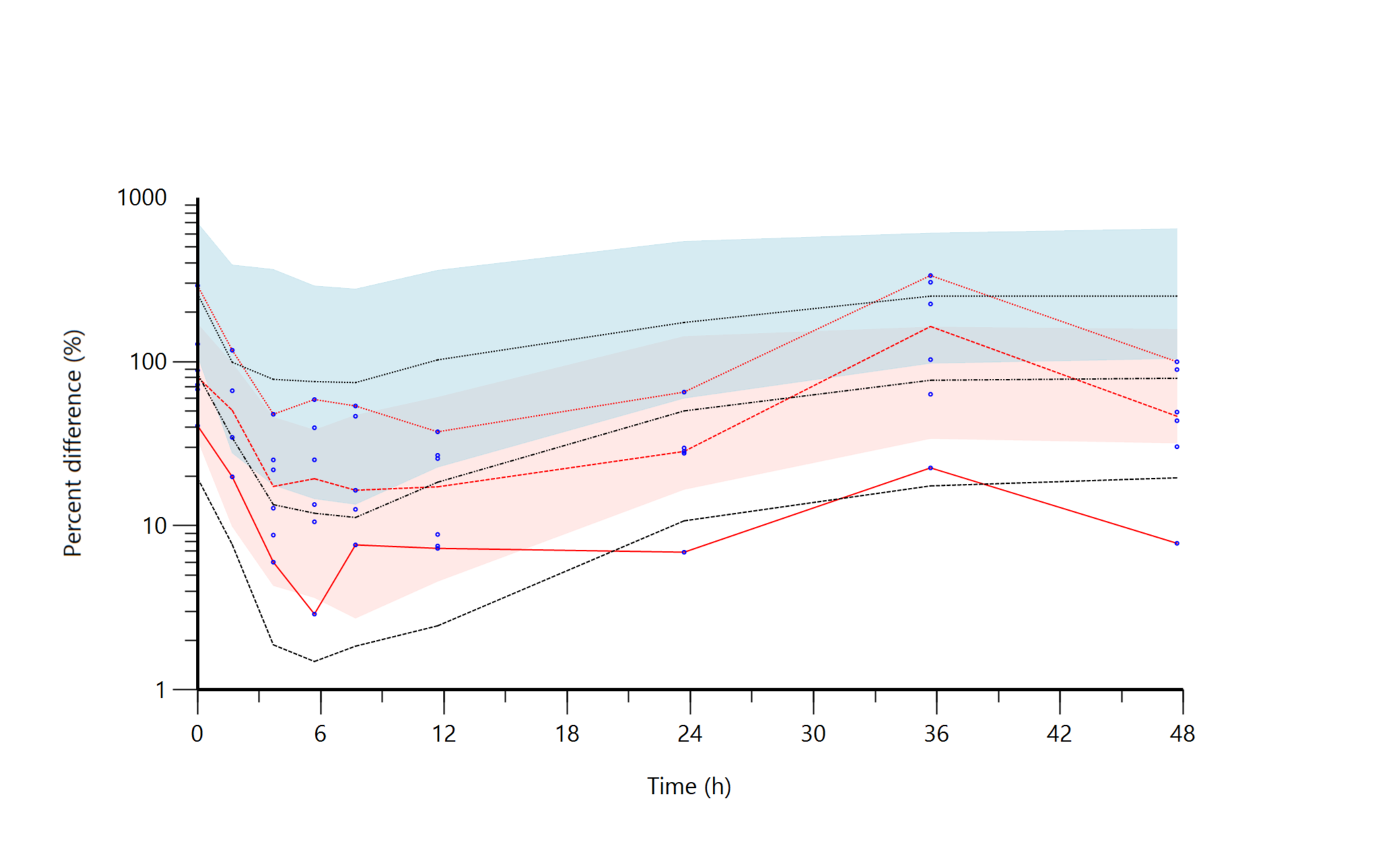


**Figure S3.** Visual predictive check (VPC) of the final model for percent difference in PGE2 following administration of ketoprofen, compared to untreated, castrated and tail docked control piglets (using 300 replicates). Observed PGE2 percentages are depicted by the open circles. The 5th, 50th and 95th percentiles of the observed concentrations are represented by the red lines. The 5th, 50th and 95th percentiles of the predicted concentrations are represented by the black dashed lines. The 95% confidence intervals (CI) for the predicted 5th and 95th percentiles are represented by the blue shaded regions. The 95% CI for the predicted 50th percentile is represented by the red shaded region.


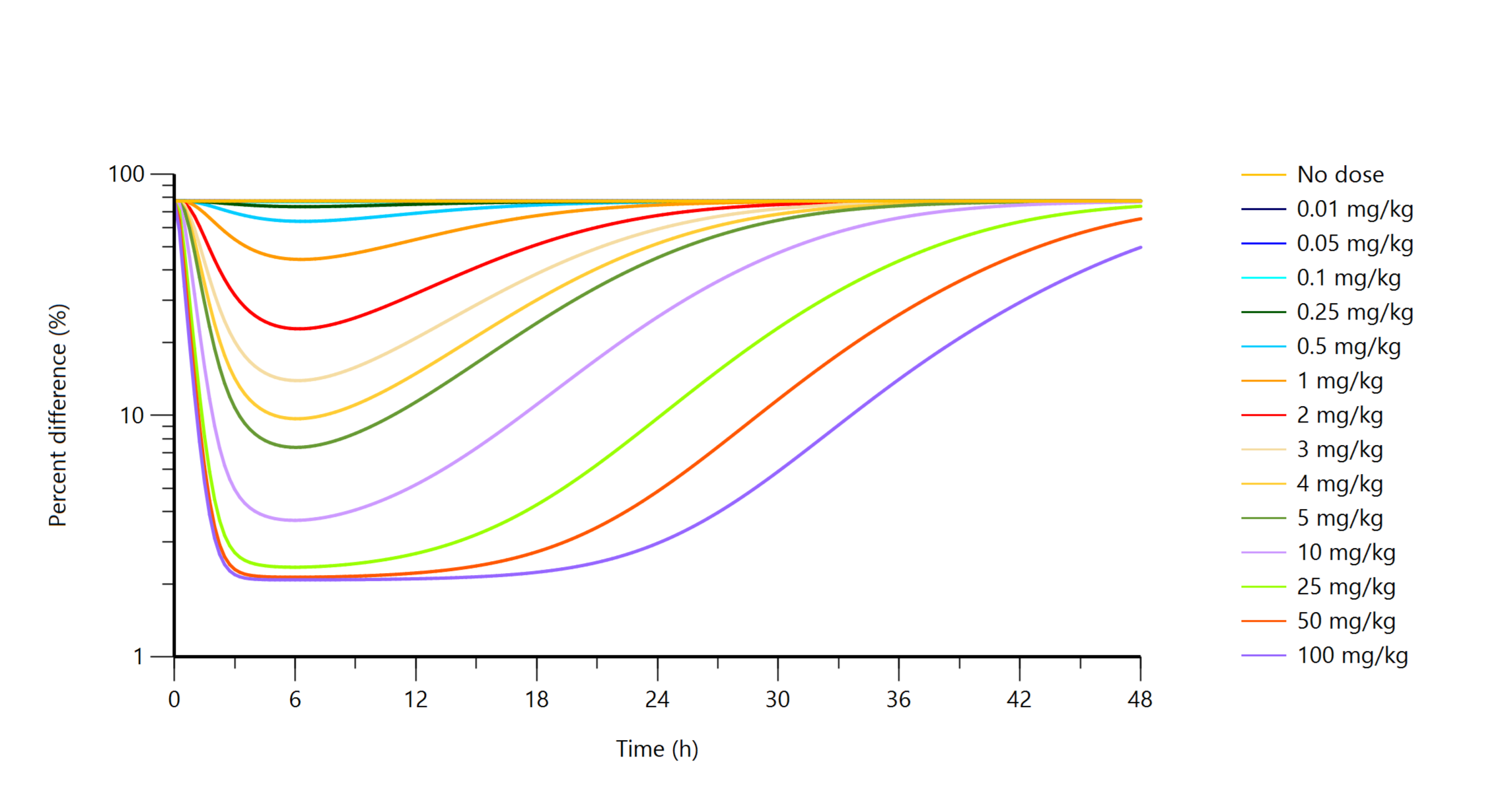
**Figure S4.** Simulated time proﬁle of PGE2 for 15 single intramuscular dose administrations of ketoprofen to piglets undergoing castration and tail-docking.


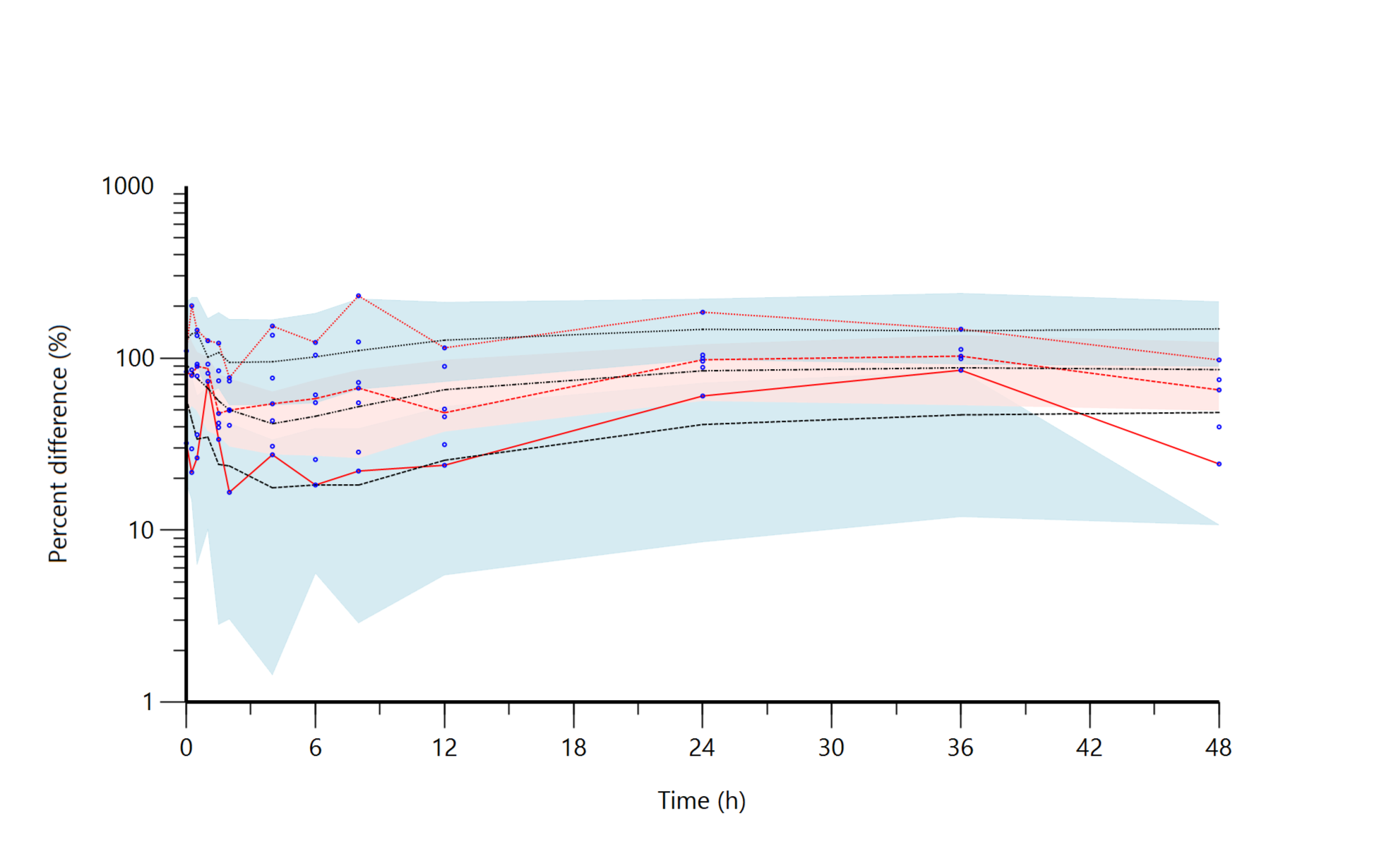


**Figure S5.** Visual predictive check (VPC) of the final model for percent difference in cortisol following administration of ketoprofen, compared to untreated, castrated and tail docked control piglets (using 300 replicates). Observed cortisol percentages are depicted by the open circles. The 5th, 50th and 95th percentiles of the observed concentrations are represented by the red lines. The 5th, 50th and 95th percentiles of the predicted concentrations are represented by the black dashed lines. The 95% confidence intervals (CI) for the predicted 5th and 95th percentiles are represented by the blue shaded regions. The 95% CI for the predicted 50th percentile is represented by the red shaded region.


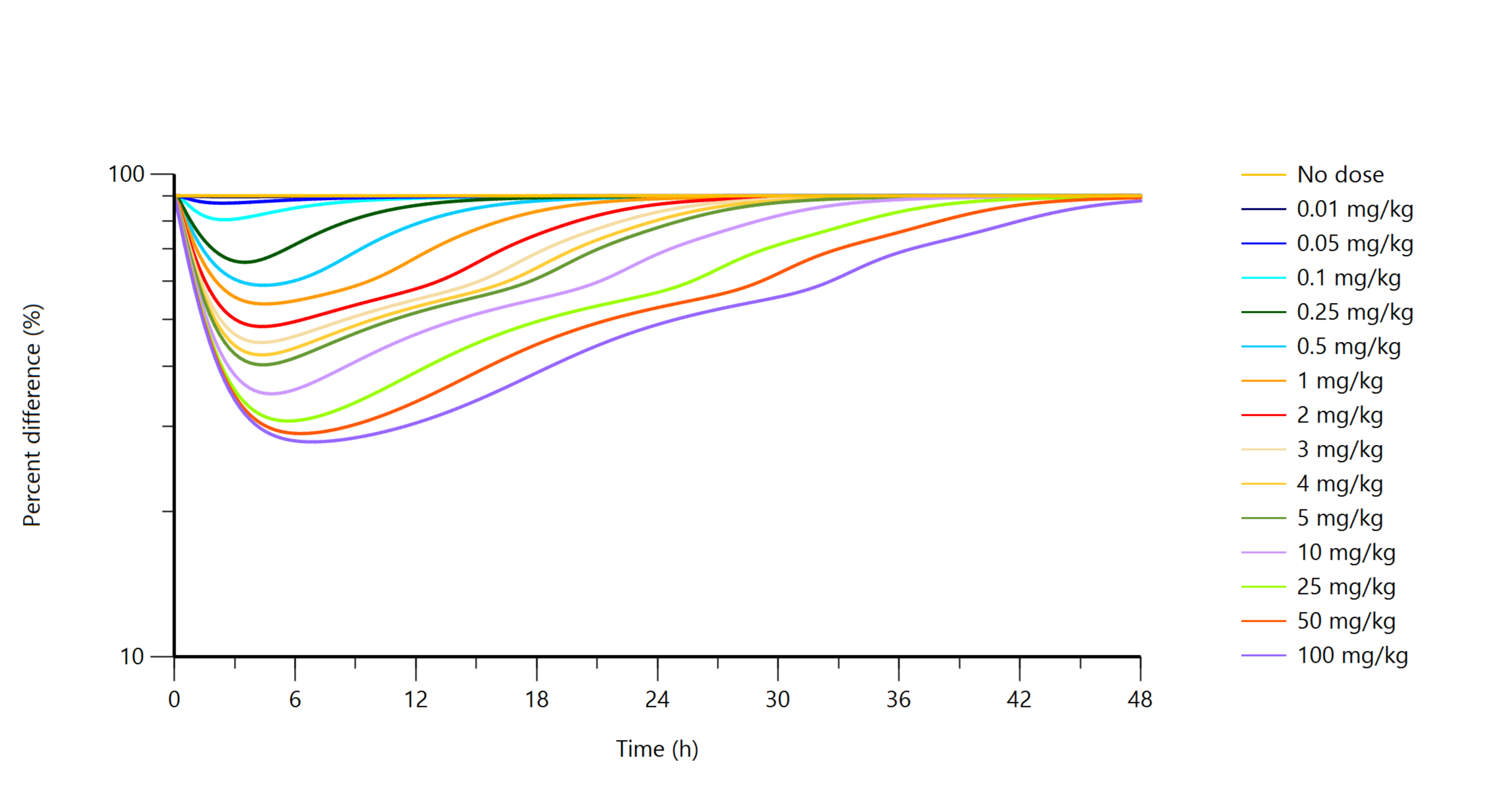


**Figure S6.** Simulated time proﬁle of cortisol for 15 single intramuscular dose administrations of ketoprofen to piglets undergoing castration and tail-docking.


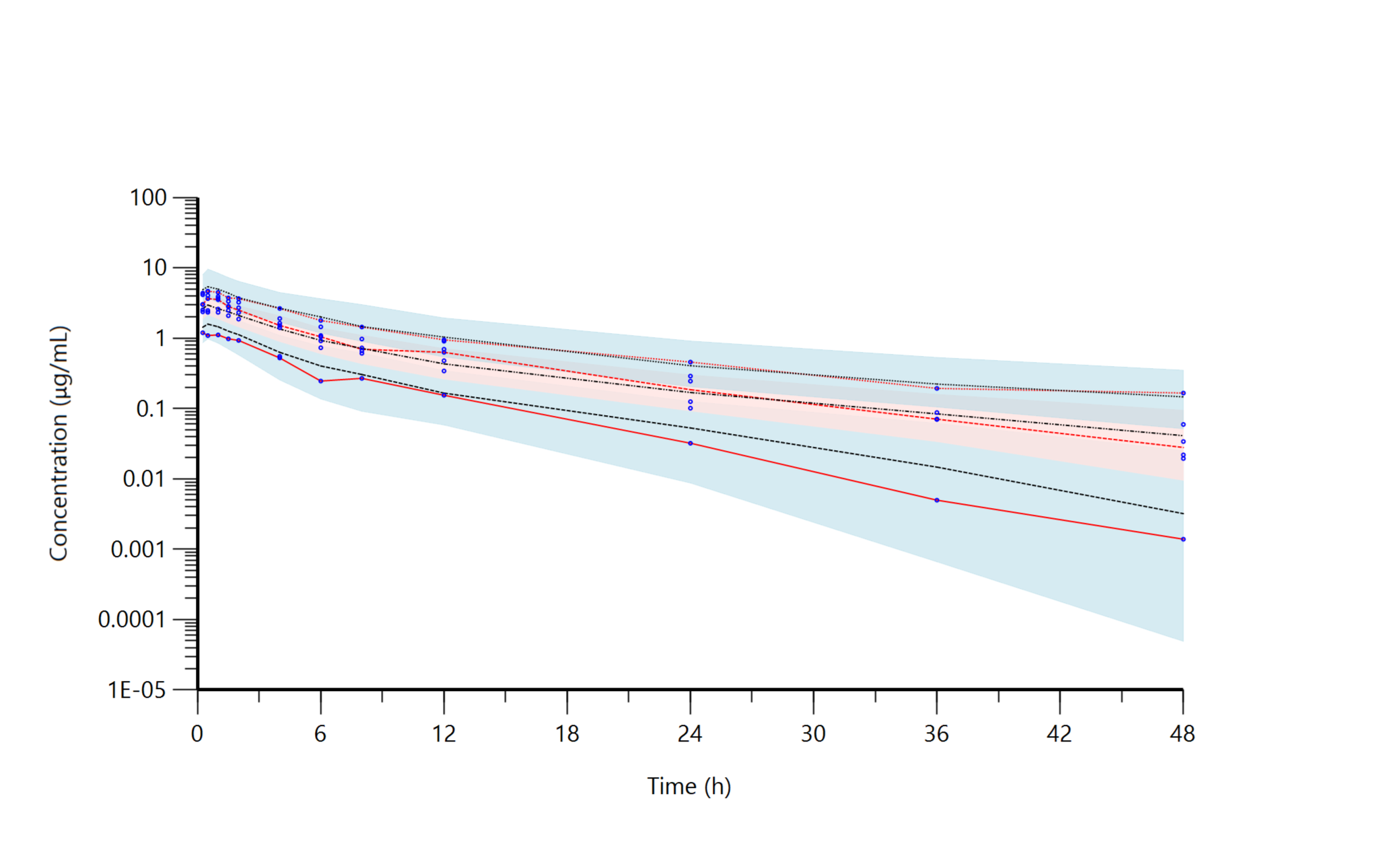


**Figure S7.** Visual predictive check (VPC) of the final model for flunixin in plasma (using 300 replicates). Observed plasma concentrations are depicted by the open circles. The 5th, 50th and 95th percentiles of the observed concentrations are represented by the red lines. The 5th, 50th and 95th percentiles of the predicted concentrations are represented by the black dashed lines. The 95% confidence intervals (CI) for the predicted 5th and 95th percentiles are represented by the blue shaded regions. The 95% CI for the predicted 50th percentile is represented by the red shaded region.


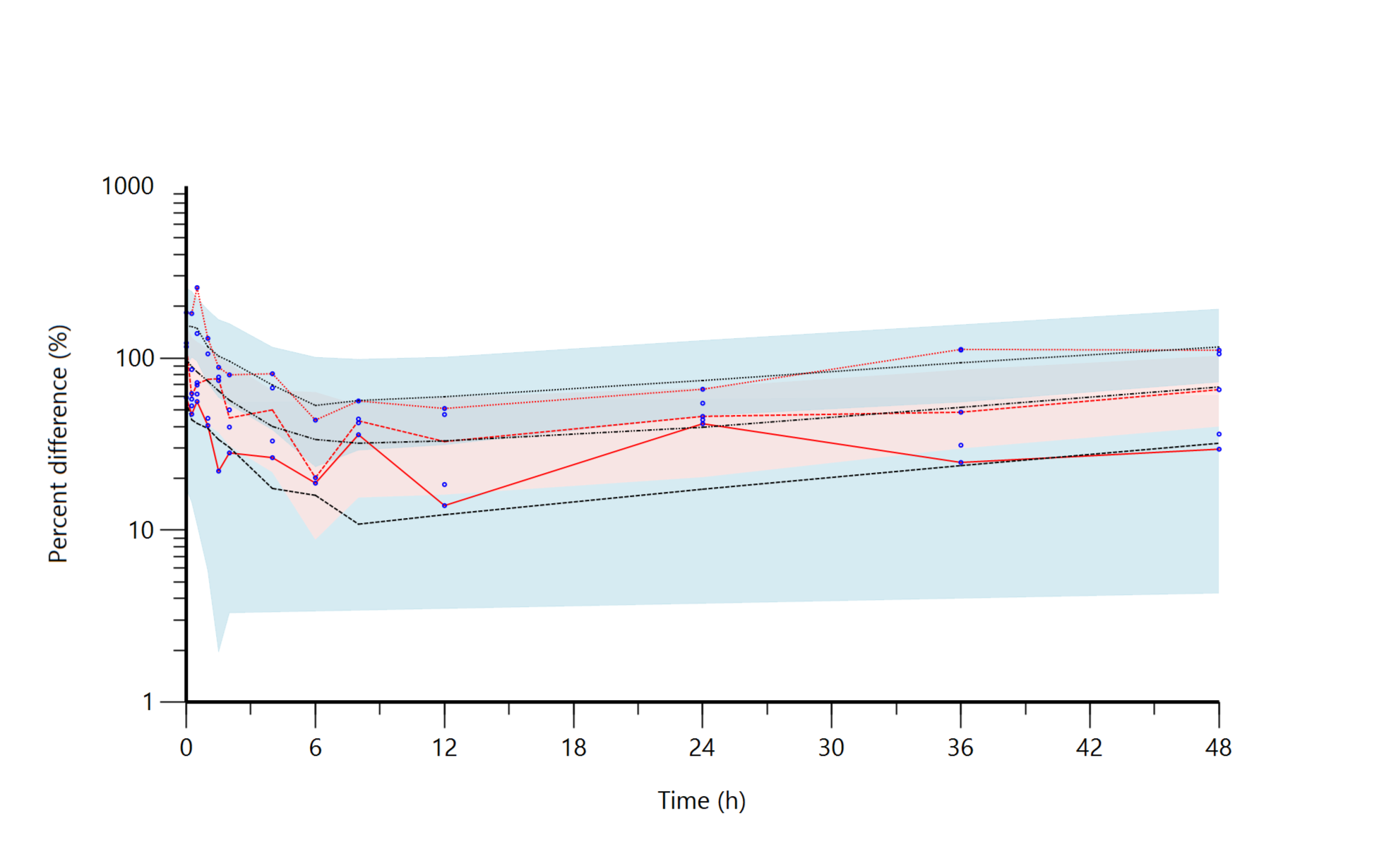
 **Figure S8.** Visual predictive check (VPC) of the final model for percent difference in cortisol following administration of flunixin, compared to untreated, castrated and tail docked control piglets (using 300 replicates). Observed cortisol percentages are depicted by the open circles. The 5th, 50th and 95th percentiles of the observed concentrations are represented by the red lines. The 5th, 50th and 95th percentiles of the predicted concentrations are represented by the black dashed lines. The 95% confidence intervals (CI) for the predicted 5th and 95th percentiles are represented by the blue shaded regions. The 95% CI for the predicted 50th percentile is represented by the red shaded region.


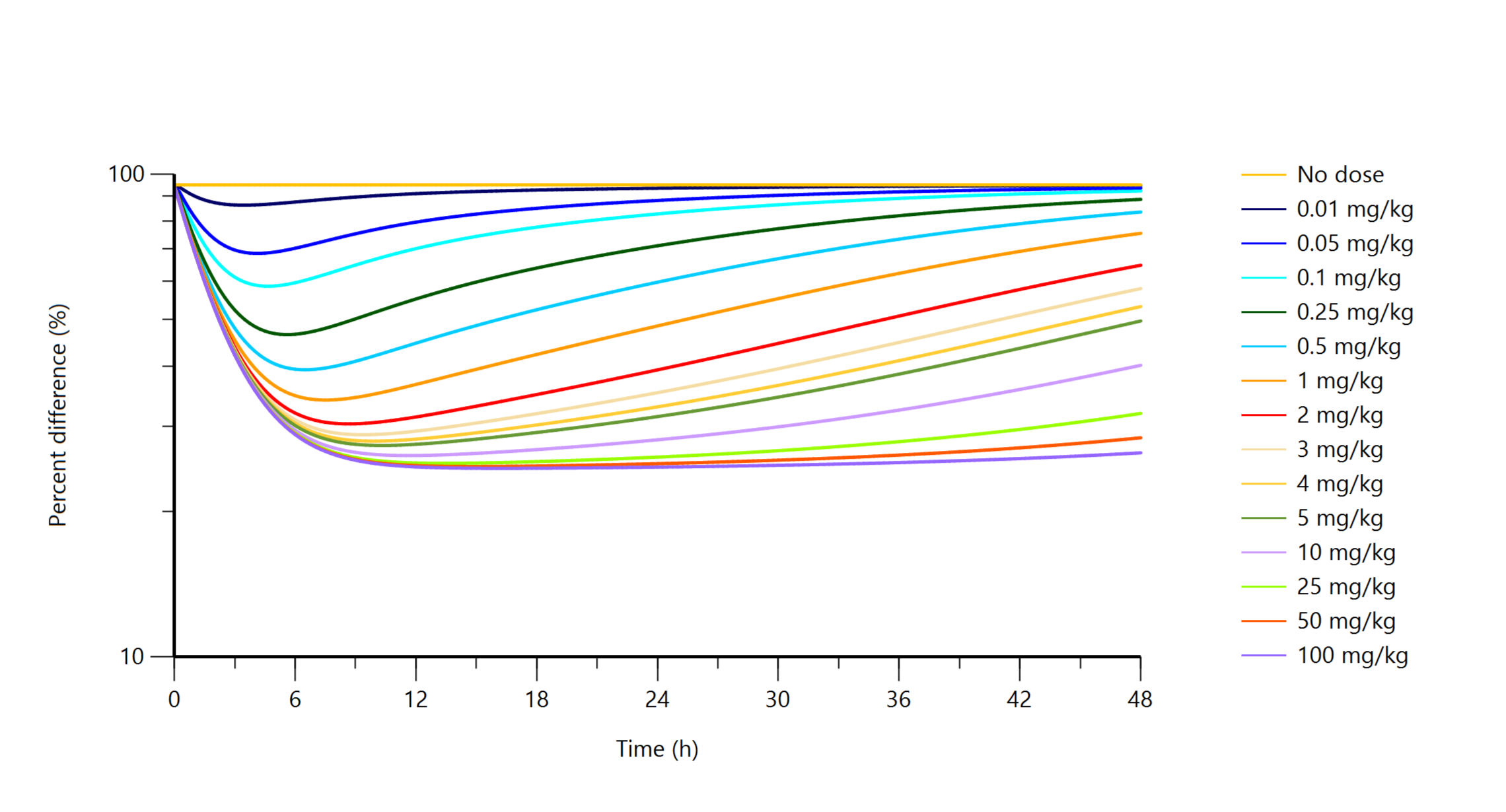


**Figure S9.** Simulated time proﬁle of cortisol for 15 single intramuscular dose administrations of flunixin to piglets undergoing castration and tail-docking.
